# *De novo* genome assembly and population genomics of a shrub tree *Barthea barthei* (Hance) krass provide insights into the adaptive color variations

**DOI:** 10.3389/fpls.2024.1365686

**Published:** 2024-05-01

**Authors:** Weicheng Huang, Bin Xu, Wei Guo, Zecheng Huang, Yongquan Li, Wei Wu

**Affiliations:** ^1^ College of Horticulture and Landscape Architecture, Zhongkai University of Agriculture and Engineering, Guangzhou, China; ^2^ South China Botanical Garden, Chinese Academy of Science, Guangzhou, China; ^3^ Guangdong Provincial Key Laboratory of Silviculture, Protection and Utilization, Guangdong Academy of Forestry, Guangzhou, China

**Keywords:** flower color divergence, ecological adaptation, anthocyanin biosynthesis, whole-genome duplication, natural selection, *Barthea barthei*

## Abstract

Flower color is a classic example of an ecologically important trait under selection in plants. Understanding the genetic mechanisms underlying shifts in flower color can provide key insights into ecological speciation. In this study, we investigated the genetic basis of flower color divergence in *Barthea barthei*, a shrub tree species exhibiting natural variation in flower color. We assembled a high-quality genome assembly for *B. barthei* with a contig N50 of 2.39 Mb and a scaffold N50 of 16.21 Mb. The assembly was annotated with 46,430 protein-coding genes and 1,560 non-coding RNAs. Genome synteny analysis revealed two recent tetraploidization events in *B. barthei*, estimated to have occurred at approximately 17 and 63 million years ago. These tetraploidization events resulted in massive duplicated gene content, with over 70% of genes retained in collinear blocks. Gene family members of the core regulators of the MBW complex were significantly expanded in *B. barthei* compared to Arabidopsis, suggesting that these duplications may have provided raw genetic material for the evolution of novel regulatory interactions and the diversification of anthocyanin pigmentation. Transcriptome profiling of *B. barthei* flowers revealed differential expression of 9 transcription factors related to anthocyanin biosynthesis between the two ecotypes. Six of these differentially expressed transcription factors were identified as high-confidence candidates for adaptive evolution based on positive selection signals. This study provides insights into the genetic basis of flower color divergence and the evolutionary mechanisms underlying ecological adaptation in plants.

## Introduction

Flowers are the most distinctive organs of angiosperms and play an essential role in their extensive diversification (Theissen and Melzer, 2007; Specht and Bartlett, 2009). As the reproductive structures, flowers facilitate outcrossing and gene flow through interactions with pollinators, and the myriad shapes, colors, and scents of flowers represent evolutionary adaptations to attract different suites of pollinators (Barrett, 2008). Flower petal color is a classic example of an ecologically important trait under selection in plants. Pigments that produce the diverse palette of flower colors play a key role in pollinator attraction by providing cues for different pollinator species (Fenster et al., 2004). For instance, flowers pollinated by bees and flies tend to have brighter yellow or purple colors, while bird-pollinated flowers exhibit more vivid reds. Petal color can also influence heat capture, UV protection, and herbivore avoidance (Fairnie et al., 2022). From an evolutionary perspective, shifts in flower color are a common way for plant populations to adapt to new pollinator environments or other selection pressures (Hopkins and Rausher, 2012; Koski and Galloway, 2020). Understanding the genetic mechanisms underlying changes in flower color can provide key insights into ecological speciation, pollinator-mediated selection, and the evolution of species interactions.

So far, the contributions of flower color transitions to ecological speciation have been extensively studied in the monkeyflower genus *Mimulus* (Yuan et al., 2013). A well-characterized example is the *Mimulus lewisii* complex, which contains the pink-flowered *M. lewisii* pollinated by bees and the red-flowered *M. cardinalis* pollinated by hummingbirds. The pollinator specificity contributes to premating reproductive isolation between the two sister species (Schemske and Bradshaw, 1999). In *Mimulus*, flower colors are determined by two major pigment types - anthocyanins, which are responsible for pink/purple hues, and carotenoids, which produce yellow colors (Streisfeld and Kohn, 2005; Cooley and Willis, 2009). The shift from pink flowers in *M. lewisii* to red flowers in *M. cardinalis* was enabled by increased levels of both anthocyanins and carotenoids. In contrast, the pale pink flowers of *M. lewisii* contain low amounts of anthocyanins and lack carotenoids. The differential regulation of these two pigment classes contributes to the divergent flower colors between *M. lewisii* and *M. cardinalis* (Yuan et al., 2013). Past researches have characterized the core enzymes involved in the biosynthesis of anthocyanins and carotenoids across different species. For example, the anthocyanin biosynthetic pathway (ABP) contains at least six enzyme-encoding genes: *Chalcone synthase* (*CHS*), *Chalcone isomerase* (*CHI*), *Flavonoid 3-hydroxylase* (*F3H*), *Dihydroflavonol 4-reductas*e (*DFR*), *Anthocyanidin synthase* (*ANS*) and *UDP-3-O-glucosyltransferases* (*UF3GT*). In diverse plant species including *Petunia*, *Meconopsis*, *Lysimachia*, *Dendrobium*, *Mimulus*, *Antirrhinum*, these ABP genes were identified to be coordinately activated by highly conserved MYB-bHLH-WD40 (MBW) protein complex (Khongkhuntian, 2012; Albert et al., 2014; Ou et al., 2024; Yuan et al., 2014; Sánchez-Cabrera et al., 2021; Wang et al., 2022). The activating MYB proteins fall into subgroup 6 within the R2R3-MYB family, while the bHLH activators classify into subgroup IIIf of the bHLH transcription factor family (Stracke et al., 2001; Feller et al., 2011). In addition to regulating anthocyanin biosynthesis, the conserved MBW complex plays diverse developmental roles in Arabidopsis (Ramsay and Glover, 2005). Hence, identification of the ABP genes and their MBW regulators underlying the flower color in non-model organism has been an intricate task. So far, diverse strategies have been utilized. For instance, isolating mutants with altered flower pigmentation has identified many structural and regulatory genes involved in anthocyanin biosynthesis and other pigment pathways. Examples include ‘*ros’* mutants in *Antirrhinum* (Schwinn et al., 2006) and ‘*boo’* mutants in *M. lewisii* (Yuan et al., 2014). Fine-mapping and transgenic experiments showed that natural variants of the R3 MYB repressor *ROI1* control differential floral anthocyanin accumulation between *M. lewisii* and *M. cardinalis* (Yuan et al., 2013). In recent years, the newly developed tools such as transcription factor binding assays (Mao et al., 2021), CRISPR/Cas9 mutagenesis (Tu et al., 2022) have been used to pinpoint the cause loci for flower color variations. With more and more available genome resources and molecular tools, the chances of deeply understanding the precise molecular bases and developmental mechanisms of flower color diversification were more tractable.

**Figure 1:**
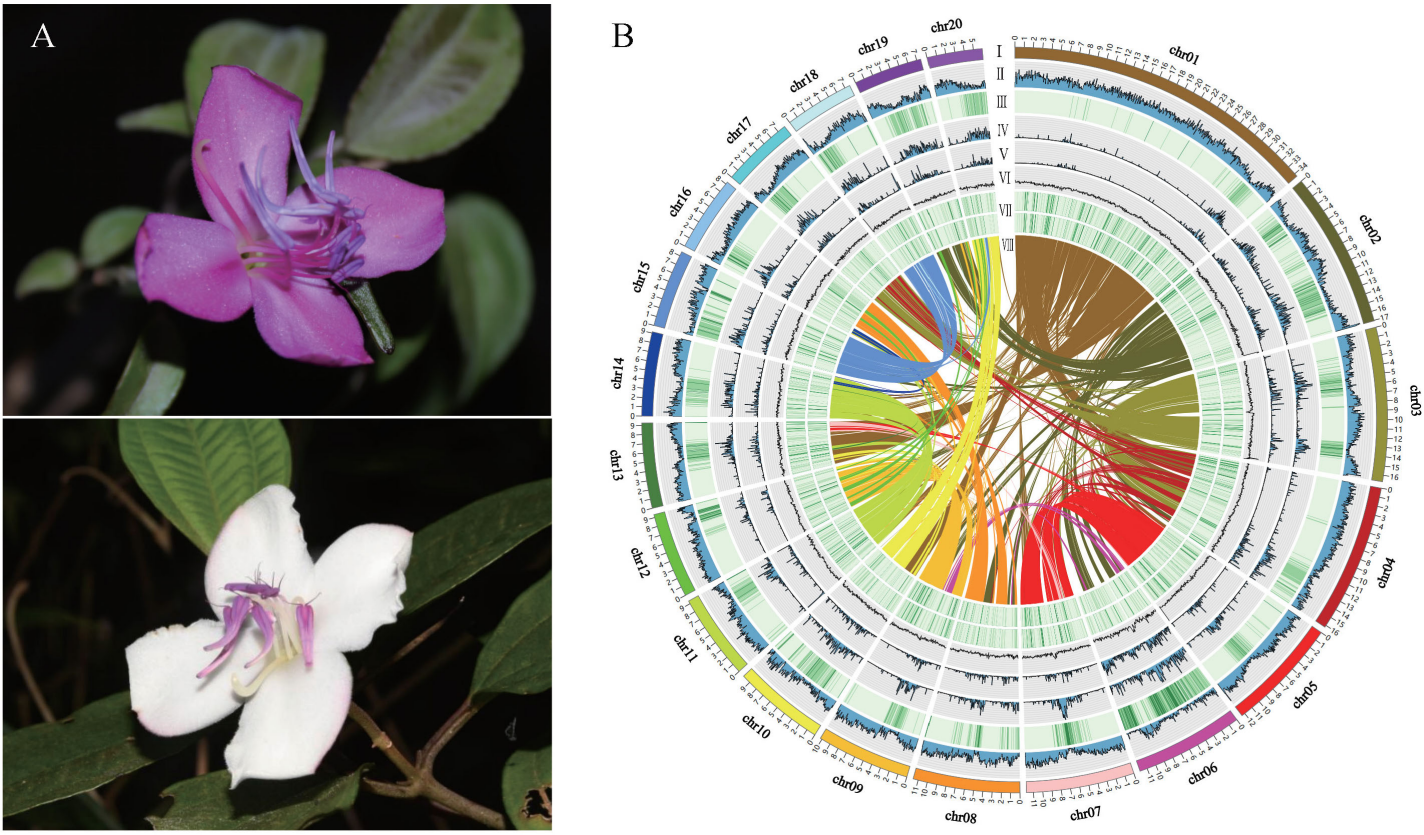
Flower morphology, whole genome features, and synteny of *Barthea barthei.* **(A)** White/purple-red color flower of *B. barthei*. **(B)** Overview of *B. barthei* draft genome assembly: (I) The 20 pseudomolecules ranged in size from 34.35 Mb to 5.96 Mb, with concentric circles shown using a window size of 100 kb; (II-VIII) represents the distribution of gene density, repeat density, TE density, Class I of TE density, Class II of TE density, gene expression level, and syntenic blocks, respectively.

In our study, the shrub tree *Barthea barthei*, a monotypic species in the Melastomaceae family, was distributed in subtropical and tropical China (Chen, 1984; Chen and Renner, 2007). This species inhabits hillsides, mountain valleys or mountain tops, in sparse or dense forests ranging from 200m to 2000m in altitude. Two varieties were previously recognized but not supported by population genetics analysis (Huang et al., 2017). Unlike the genus *Melastoma*, in which only red or pink flowers exist, there are two flower color variations, white and pink/purple, in *B. barthei*, making it a promising gardening tree for the future (**Figure 1A**). The pink/purple flowers are often observed in open areas such as mountain tops, where they are subject to strong light, UV radiation, drastic moisture changes, and winds. In contrast, the white flowers are found in understory forests, where there are mild light conditions and stable moisture and winds. Therefore, we designated them as the pink/purple ecotype and white ecotype, respectively. Hence, the natural variations of flower colors in the two eco-types provide an ideal system for dissecting the underlying molecular mechanism, especially the homologs of ABP and their MBW regulators in *B. barthei*. To achieve this goal, we assembled a high-quality genome assembly for it, and conducted transcriptome profiling comparisons. In addition, we tried to discern the genome selection signals on the flower color variation between the two eco-types. Our study will deepen our understanding of ecological adaptation in natural populations.

## Materials and methods

### Plant materials, library construction, and sequencing

One individual plant used for *de novo* genome assembly was transplanted from Wutongshan National Forest Park in Shenzhen, Guangdong province, China. A total of 31 individua plants from five populations with purple red and white petals were also sampled for genome resequencing (**Supplementary Table S1**). Fresh leaves/petals used for the genome assembly or transcriptome sequencing were sampled and frozen immediately using liquid nitrogen until DNA/RNA extractions at -80°C, and sillical-dried leaves were used for genome resequencing. Using modified cetyltrimethylammonium bromide (CTAB) (Stacey and Isaac, 1994), total DNA/RNAs were extracted. Prior to library construction and sequencing, we firstly assessed the amounts and integrity of nuclear acids using electrophoresis with 1.2% agarose gels, then evaluated using Nanodrop spectrophotometer (Thermo Fisher Scientific, USA). Qualified DNAs were subject to library constructions with insertion size of 20kb or 350bp respectively, and sequenced on Pacific Biosciences (PacBio) Sequel II platform or Illumina’s Hiseq 2000 platform respectively. The Hi-C libraries were with enzyme *Dpn*II and sequenced on the Illumina HiSeq XTen platform in paired-end mode.

### Genome assembly and evaluation of the assembly quality

The genome size of *B. barthei* was estimated based on 21-mer frequency distribution using GenomeScope2 software (Vurture et al., 2017; Ranallo-Benavidez et al., 2020). Raw PacBio subreads were corrected and assembled using the FALCON assembler v0.03 with default parameters (seed_coverage = 20, length_cutoff = -1). The initial assemblies consisting of primary contigs (p-contigs) were polished using NextPolish v1.2.2 software (Hu et al., 2020) using both long subreads and short Illumina reads. With designation of haploid chromosome number of 20 for *B. barthei* (referencing to the closely related genus *Oxyspora*), the contigs were ordered and oriented on the assumed 20 chromosomes with the valid chromatin interactions revealed by the Hi-C reads using the package ALLHIC v0.9.8 (Zhang et al., 2019b). We assessed the quality of genome assembly using three different strategies. First, we estimated the mapping rate for genome resequencing reads and transcripts assembled from RNA-sequencing to the assembly using bowtie2 v2.4.2 (Langmead and Salzberg, 2012) and HISAT2 v2.2.1 (Kim et al., 2019) respectively; Secondly, we evaluated the completeness and contingency with the coverage of core conserved Eukaryotic gene sets using BUSCO (Benchmarking Universal Single-Copy Orthologs, v5.1.2) (Seppey et al., 2019); Thirdly, we calculated the value of LTR Assembly Index (LAI) (Ou et al., 2018).

### Repeat prediction and non-coding RNA annotation

To identify and mask repetitive elements in the *B. barthei* genome assembly, we first constructed a *de novo* species-specific repeat library using RepeatModeler v1.0.11 (Flynn et al., 2020). This library was combined with existing repeat databases Dfam 3.0 (Wheeler et al., 2013) and RepBase (Bao et al., 2015) to generate a comprehensive custom library. The *B. barthei* genome assembly was then masked using RepeatMasker v4.0.9 (Tarailo-Graovac and Chen, 2009) against the custom repeat library to identify interspersed and tandem repeat sequences.

Using INFERNAL v1.1.2 software (Nawrocki and Eddy, 2013) and tRNAscan-SE v2.0.8 software searching against the RNA family database (RFAM v12.0) (Griffiths-Jones et al., 2003) with default parameters. (Chan and Lowe, 2019) (http://lowelab.ucsc.edu/tRNAscan-SE/), the noncoding RNAs (including tRNA, rRNA, miRNA, snRNA) were predicted.

### Gene prediction and functional annotation

For protein-encoding gene prediction, we used the pipeline GETA v2.4.6 (https://github.com/chenlianfu/geta) with combined homolog-based and *de novo* approaches. For this pipeline, transcripts from the RNA-sequencing of *B. barthei* or proteins from related species including *Eucalyptus grandis*, *Arabidopsis thaliana*, *Cirtus sinensis*, *Gossypium raimondii*, *Medicago truncatula*, *Populus trichocarpa*, *Vitis vinifera*, *Cucumis melo*, *Melastoma candidium*, *Prunus persica* and *Mimulus guttatus* (Details of sources in **Supplementary Table S1**), were aligned to the genome assembly using HISAT2 v2.2.1 (Kim et al., 2019) or Genewise v2.4.1 (Birney et al., 2004), respectively. Then credible, complete gene structures were obtained by using this homology approach. With subsets of these gene models, several rounds of gene model training were implemented in the package Augustus v3.2.3 (Stanke and Morgenstern, 2005). Using the optimized gene models, *ab initio* prediction using the same package was conducted by the hints of intron, CDS, exon, start and stop codon. Finally, all predicted genes were integrated and filtered with convincing domain evidences from Pfam v35.0 database (El-Gebali et al., 2019). Function annotation of the predicted protein-coding genes was performed by searching against several protein databases using BLASTP, including the NR protein database (Sayers et al., 2021), Swiss-Prot (Gasteiger et al., 2001), COG (Tatusov et al., 2003), and eggNOG (http://eggnogdb.embl.de/) (Huerta-Cepas et al., 2019). Additionally, motifs and domains were annotated by searching against the InterPro v5.3.46 (Blum et al., 2021) and Pfam databases using Interproscan v4.7 (Quevillon et al., 2005) and Hmmer v3.3.2 (Mistry et al., 2013), respectively. Gene Ontology (GO) terms were assigned by integrating the InterPro and eggNOG annotations. The KEGG (Kyoto Encyclopedia of Genes and Genomes) annotations were obtained by using the KAAS web tool (Moriya et al., 2007).

### Gene family evolution and phylogenomic analysis

Using OrthoFinder v2.5.4 (Emms and Kelly, 2019) with an inflation value of 1.5, gene families between *B. barthei* and 11 other plant genomes were identified (**Supplementary Table S1**). Gene family expansions and contractions were detected using the CAFE program v4.2.1 (De Bie et al., 2006) with default parameters. For each single or low copy nuclear gene (copy number less than 3), their protein sequences from each species were aligned with MAFFT v7.0 (Katoh and Standley, 2013), then their corresponding coding sequences were aligned to the protein alignments with no gaps and no mismatch using the package PAL2NAL v14 (Suyama et al., 2006). Next, both the concatenation and coalescent approaches used for the phylogeny construction. For the concatenation approaches, the CDS alignments for the single copy nuclear genes were concatenated into a supermatrix, and subject to substitution model test using the package jModelTest2 (Darriba et al., 2012) with the Akaike information criterion. Following the identified substitution mode and using *Vitis vinifera* as outgroup, a maximum-likelihood tree was constructed using RaxML v8.2.10 (Stamatakis, 2014) with 1000 bootstrap replicates. For the coalescent approach, Maximum likelihood estimation of gene trees for low-copy genes were constructed by RaxML, and the trees files were used to infer the species tree using ASTRAL v5.6.1 (Yin et al., 2019).

Divergence times among the 12 species were estimated using the MCMCTREE program in PAML v4.9 (Yang, 2007). The analysis was run with the following parameters: burn-in = 2000000, sampfreq = 100 and nsample = 100000. Two calibration points were used to date the divergences: one between Arabidopsis and Populus (82.8-127.2 million years ago, Mya) based on Clarke et al. (2011), and another between Myrtaceae and Melastomataceae (101.0-116.0 Mya) based on Berger et al. (2016).

### Genome synteny and whole genome duplication

Using the program MCScanX (Wang et al., 2012), the protein sequences of *Eucalyptus grandis*, *Vitis vinifera* and *B. barthei* were implemented both self-blast and reciprocal blast using BLASTp v2.10.1 with E-values < 1e-5, and the top 5 hits of each query were retained to determine intra/inter genomic collinear blocks. For each paralogous gene pair within these collinear blocks, the number of nonsynonymous substitutions per nonsynonymous site (Ka) and synonymous nucleotide substitutions site (Ks) were calculated using the Nei-Gojobori algorithm as implemented in a built-in perl scripts of MCScanX (add_ka_and_ks_to_collinearity.pl). If Ka/Ks > 1, the collinear genes were likely to have potentially experienced positive selection. The genome collinearities within each species and between species were visualized using WGDI v0.5.2 (Sun et al., 2022). In addition, we used the median Ks value of each collinear genomic region to infer the time of the WGD event. The kernel smoothing density function was used to generate Ks distribution curves. Gaussian multi-peak fitting in WGDI was then utilized to further resolve peaks in the distribution curves. These peaks correspond to hypothesized ancestral genome duplication events. By comparing the timing of ancestral duplication events across species, we aimed to date the lineage-specific WGD event.

### Identification of the anthocyanin biosynthesis-related transcription factors in the genome assembly of *B. barthei*

We downloaded the protein sequences of MYBs, bHLH, and WD40 in the species Arabidopsis from PlantTFDB 4.0 database (Jin et al., 2017). For each transcription factor families, they were used to query about the protein sequences of *B. barthei* with an E-value cut-off of 1e-10. Additionally, profile hidden Markov models (HMMs) of the DNA-binding domains for each TF family (PF000249 for MYB, PF00010 for bHLH, and PF00400 for WD40) were downloaded from Pfam and searched against *B. barthei* protein sequences using HMMER v3.3.2 (Finn et al., 2011). The BLAST and HMMER results were intersected to identify common elements, representing putative homologs of the queried TF families in *B. barthei*. To verify the reliability of the intersected results, the completeness of the TF gene domains was analyzed using Pfam and the NCBI Conserved Domain Database (CDD). For phylogenetic analysis, the amino acid sequences of each TF family from *A. thaliana* and *B. barthei* were aligned using MAFFT v7.453 (Katoh and Standley, 2013). Phylogenetic trees were constructed using the maximum likelihood (ML) method with 1000 bootstrap replicates in FastTree v2.1.10 (Price et al., 2009). The Jones-Taylor-Thornton (JTT) model of amino acid substitution was used, and rates among sites were modeled using a gamma distribution.

### Transcriptomic changes underlying flower color differences between purple-red and white eco-types

The raw RNAseq reads from six individuals, each with either purple or white flowers and represented by three biological replicates, were processed to remove contaminated and low-quality reads using fastp v0.20.1 with default parameters (Chen et al., 2018). The resulting clean reads were then mapped to the reference genome using HISAT2. Gene-level transcript quantification was performed with featureCounts (Liao et al., 2014). Differential expression analysis between the two groups was conducted using DESeq2 v3.1.3 (Love et al., 2014) with FDR-adjusted p-value < 0.05 and absolute log2 fold change ≥ 2. Heatmap of expression profiles were generated using TBtools (Chen et al., 2020). Protein-protein interaction networks were constructed for the identified BbbMYB, BbbHLH, and BbWD40 candidate genes using STRING v11.5 (https://cn.string-db.org/). Only high-confidence interactions with a minimum required interaction score ≥ 0.9 were included in the networks.

### Population genomics analysis for the two ecotypes of *B. barthei*

#### Reads mapping and variants calling

The raw pair-end reads of 31 *B. barthei* accessions were resequenced with at least 10-fold depth (**Supplementary Table S1**). The raw reads were then trimmed to remove adapters and low-quality bases using fastp. The clean reads were mapped to the *B. barthei* reference genome using BWA v0.7.17 (Li and Durbin, 2010) with default parameters. The mapped reads were sorted and duplicate reads were removed using SAMtools v1.10 (Etherington et al., 2015). Variants were called using the Realigner Target Creator and Indel Realigner programs from the GATK package v3.8 (DePristo et al., 2011). The GATK HaplotypeCaller was used to estimate SNPs and indels with default parameters. Low-quality SNPs were filtered from the raw VCF dataset based on the following criteria: QD < 2.0, MQ < 40.0, FS > 60.0, SOR > 3.0, MQRankSum < -12.5, or ReadPosRankSum < -8.0. Low-quality indels were similarly filtered using the criteria: QD < 2.0, FS > 200.0, SOR > 10.0, MQRankSum < -12.5, or ReadPosRankSum < -8.0. The remaining SNPs and indels were annotated using SnpEff v5.0e (Cingolani et al., 2012).

#### Population genetics analysis and demographic history inference

A neighbor-joining (NJ) phylogenetic tree was constructed from a distance matrix generated by VCF2Dis (https://github.com/BGI-shenzhen/VCF2Dis) using 31 *B. barthei* accessions. The resulting tree was visualized using FastME (http://www.atgc-montpellier.fr/fastme/) and iTOL (https://itol.embl.de/). Population structure analysis was performed using two methods. First, ancestry proportions were estimated for K ancestral populations ranging from 2 to 9 using Admixture (Alexander et al., 2009). The most likely number of populations was determined to be the K value with the lowest cross-validation error. Population stratification plots were generated using the R package pophelper v1.0.10 (Francis, 2017). Second, a principal component analysis (PCA) was conducted with PLINK v1.9 (Purcell et al., 2007) and the top three PCs were used to assign individuals into populations. To assess genome-wide linkage disequilibrium (LD) decay, the correlation coefficient (r^2) between pairs of variants was calculated as a function of distance using PopLDdecay v3.40 (Zhang et al., 2019a) with default parameters. Using package ANGSD v0.936 (Korneliussen et al., 2014) with parameters ‘-doSaf 1 -GL 2 -P 4 -minMapQ 1 -minQ 20’, the site frequency spectrum (SFS) was inferred with EM algorithm based on population SNPs from the two eco-types. The demographic history was inferred from the SFS using Stairway Plot v0.2 (Liu and Fu, 2020). The stairway plot analysis was run with 1000 bootstraps, using a mutation rate of 6.5×10^-9^ per site per generation and a generation time of 2 years. This allowed estimation of past changes in effective population size over time.

#### Genomic signatures of adaptive evolution between ecotypes of *B. barthei*

To identify genomic regions underlying natural selection, site-frequency-spectrum-based nucleotide diversity (π) along with the population differentiation-based F_ST_ (Nei and Li, 1979, Weir and Cockerham, 1984), and cross-population extended haplotype homozygosity (XP-EHH) (Sabeti et al., 2007), were calculated using a sliding window size of 20 kilobases (kb) and a step size of 2 kb respectively. Calculation of π and F_ST_ was conducted utilizing PopGenome (Pfeifer et al., 2014). The top 5% of windows based on the π ratio (π_HD/π_YC) distribution were identified as outliers. Similarly, regions exhibiting F_ST_ values within the top 5% were categorized as high- F_ST_ outliers.

XP-EHH analysis, implemented in selscan v2.0.0 (Sabeti et al., 2007), is designed to detect signals of recent positive selection by comparing differences in extended haplotype homozygosity between populations. This method evaluates the lengths of haplotypes carrying a selected allele in one population compared to another, identifying regions where these haplotypes are notably extended due to the selective pressure acting on specific genomic segments. Normalized genomics regions with absolute XP-EHH score over 2 at a p significance of 0.05 were identified, and the top 5% of these regions were filtered as being under positive selection. The high confidence gene sets under positive selection were obtained by cross validation among the three methods. Genes located within the selective sweep regions were subjected to functional enrichment analysis using KOBAS-i (Bu et al., 2021).

## Results

### Genome assembly and annotation

The genome size of *B. barthei* was estimated to be 246.44 Mb with a heterozygosity of 0.71% based on 21-mer frequency analysis (**Supplementary Figure S1**; **Supplementary Table S2**).

A total of 32.76 Gb of PacBio subreads (approximately ~130× coverage) were self-corrected and assembled into contigs using FALCON. After error correction and polishing utilizing approximately 100× Illumina paired-end reads, we obtained an assembly consisting of 250 contigs totaling 235.03 Mb with an N50 of 2.39 Mb (**Supplementary Table S3**). After clustering, anchoring and orientation using valid chromatin interactions from Hi-C data we obtained 33 scaffolds, of which 99.79% were anchored into 20 pseudochromosomes (**Figure 1B**; **Supplementary Figure S2**; **Supplementary Table S4**). The accuracy and completeness of the final genome assembly were evaluated through several quality metrics. The raw Illumina reads mapped back to the genome at 96.25% coverage, while RNA-seq reads showed 87.70-96.67% coverage (**Supplementary Table S5**). The completeness proportions compared to the core gene sets of BUSCO database was 97.1-99.6% (**Supplementary Table S6**). The LAI metric was estimated to be 13.7, consistent with a high-quality reference genome assembly (LAI 10-20). A total of 54.42 Mb (23.13%) of the genome assembly were identified to be repetitive sequences, primarily long terminal repeats (LTRs) (25.20 Mb; 10.72%) (**Supplementary Table S7**). There were 46,430 protein-encoding genes predicted, and these genes were distributed unevenly across the pseudochromosomes (**Table 1**; **Supplementary Table S8**). Out of the 46,430 predicted protein-coding genes, 42,106 (90.07%) were annotated by public databases using a threshold E-value of 0.001(**Supplementary Table S9**). In addition to protein-coding genes, 1,560 non-coding RNAs (ncRNAs) were identified, including 235 miRNAs, 240 rRNAs, 737 tRNAs, and 348 snRNAs (**Supplementary Table S10**).

**Table 1:** Summary of *Barthea barthei* genome assembly and annotations.

Characteristics	Size (proportions)
Estimated genome size (by k-mer analysis) (Mb)	246
Contig N50 (Mb)	2.39
Scaffold N50 (Mb)	11.76
Longest scaffold (Mb)	85.71
Assembled genome size (Mb)	235.03
Assembly % of genome	99.79
Repeat region % of assembly	23.13
Predicted gene models	46,430
Average coding sequence length (bp)	2,733
Average exons per gene	294

### Gene family evolution and divergence time dating

**Figure 2:**
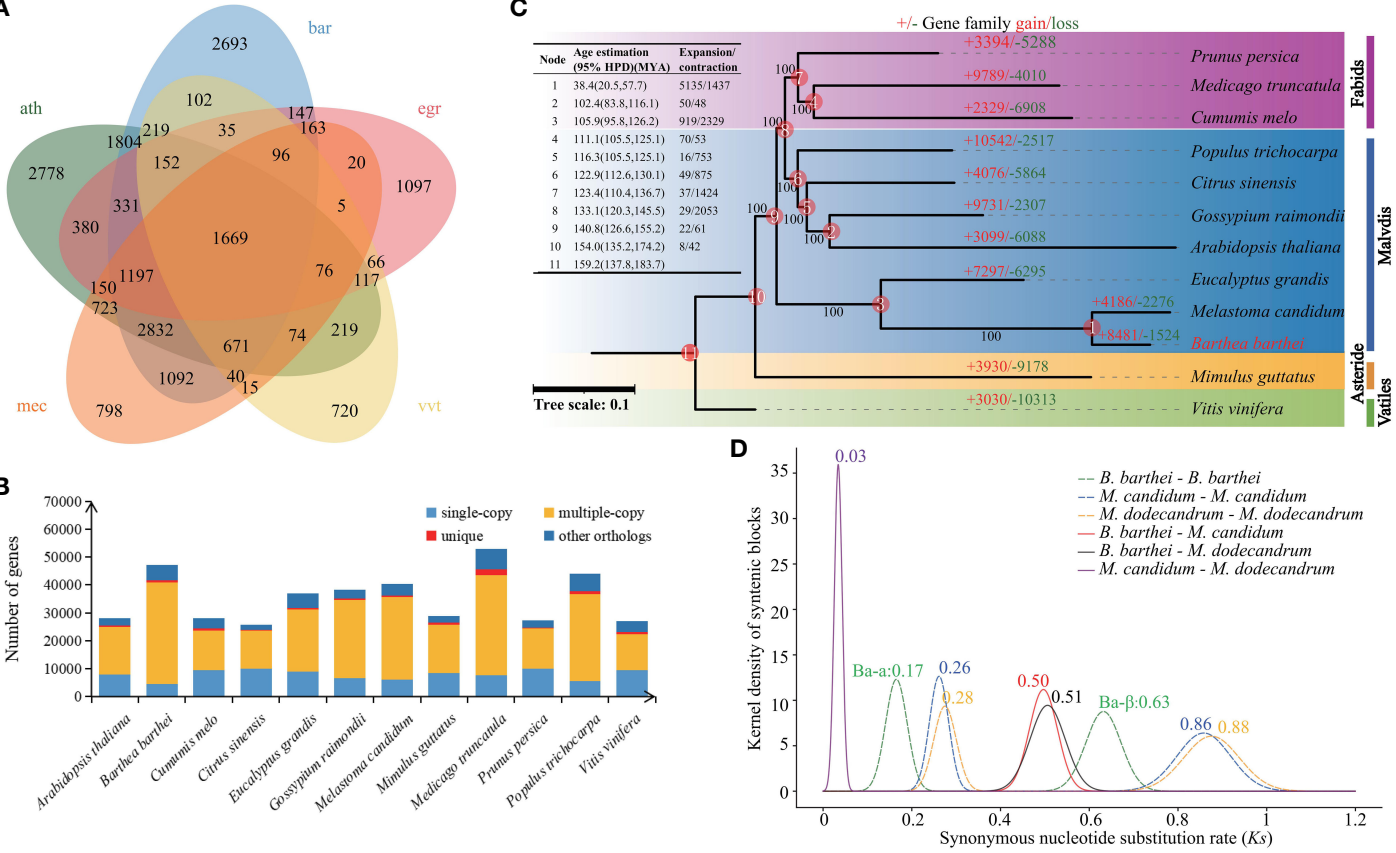
Phylogeny, gene family evolution, and whole-genome duplication history of *Barthea barthei* and related species. **(A)** Phylogenetic tree of *B. barthei* and 11 other related species, inferred from 314 single-copy orthologs. Branch lengths are proportional to the number of substitutions per site, the abbreviation for these species as following: ath: *Arabidopsis thaliana*, bar: *Barthea barthei*, egr: *Eucalyptus grandis*, mec: *Melastoma candidum*, vvt*: Vitis vinifera*; **(B)** Distribution of gene family sizes in *B. barthei* and four other representative species; **(C)** Gene family expansion and contraction events along the branches and nodes of the phylogenetic tree. Colors indicate different plant orders: Fabids, Malvids, Asterids, and Vitales. The number of gene families, orphans (single-copy gene families), and the number of predicted genes are indicated for each species; **(D)** Density distributions of Ks values for homologous gene pairs, with inferred whole-genome duplication events indicated by vertical dashed lines.

A total of 28,179 gene families comprising of 366,919 protein sequences were identified between *B. barthei* and 11 other plant species (**Figure 2B**). Among these gene families, 1,669 were shared by *B. barthei* and four other representative species (*A. thaliana*, *E. grandis*, *M. candidum* and *V. vinifera*) (**Figure 2A**). Of the 28,179 total gene families, 750 were found to be unique to *B. barthei*, containing 1,885 protein sequences. Functional enrichment analysis revealed these *B. barthei*-specific gene families were significantly enriched for 5 KEGG pathways and 155 GO terms (**Supplementary Tables S11**, **S12**). The enriched pathways included plant-pathogen interaction (KO04626, P = 3.0e-4, FDR = 0.02, Fisher’s exact test) and glucosinolates biosynthesis (KO00966, P = 3.8E-03, FDR = 3.5E-02, Fisher’s exact test), both associated with plant defense against insect pests. One notably enriched GO term was photoinhibition response (GO0010205, P = 1.3E-06, FDR = 6.6E-05, Fisher’s exact test). These genes may enable adaptation to variable light environments for *B. barthei* in subtropical/tropical forests. A maximum likelihood tree was constructed using 314 single-copy orthologs from the 12 species with *V. vinifera* as the outgroup. *B. barthei* was clustered with *M. candidum* and was sister to *Eucalyptus.* However, the branch length of *B. barthei* was about twice that of *Eucalyptus*, indicating more rapid radiation within the Melastomataceae family (**Figure 2C**; **Supplementary Figure S3**). The divergence time between *B. barthei* and *M. candidum* was estimated to be 38.4 Mya (95% HPD: 20.5-57.7 Mya). The divergence time between *Barthea* and *Eucalyptus* was 105.9 Mya (95% HPD: 100.3-114.0 Mya), dating back to the mid-Cretaceous (**Figure 2C**; **Supplementary Figure S4**).

There were 5842 gene families comprising 8481 genes expanded, and 1306 families comprising 1524 genes contracted in *B. barthei*. Of these, 51 rapidly evolving gene families were identified (**Figure 2C**; **Supplementary Table S13**). KEGG enrichment analysis of the rapidly expanded families revealed 10 significantly enriched pathways (**Supplementary Figure S5**). Notably, the phenylpropanoid biosynthesis pathway (KO00940, P = 1.2E-03, FDR = 1.3E-01) containing 16 genes was enriched. This pathway is associated with anthocyanin biosynthesis, suggesting rapid expansion may have promoted anthocyanin production in *B. barthei.*

### Genome synteny and whole-genome duplication analysis

Using MCScanX with a strict match size (s=10), 511 collinear blocks were identified in *B. barthei*, ranging from 13 to 777 gene pairs (average 62) and comprising 34,405 collinear genes (~74.12% of the gene set) (**Supplementary Figure S6**). To elucidate the WGDs in *B. barthei*, syntenic blocks were estimated between *B. barthei* and *Eucalyptus* and grape, which have known lineage-specific polyploidy events. Ratios of 2:1 for *B. barthei*: *Eucalyptus* and 4:1 for *B. barthei*: grape provide strong evidence for two tetraploidization events in *B. barthei* (**Supplementary Figures S7**–**S9**). The two WGDs occurred at *Ks* of 0.17 (designated as Ba-α) and 0.63 (designated as Ba-β) respectively (**Figure 2D**). Two WGDs were also identified in the two *Melastoma* species (*M. candidum*: Ks of 0.26, 0.86 respetively; *M. dodecandrum*: Ks of 0.28, 0.88 respectively) (**Figure 2D**). The divergence in terms of *Ks* between *Barthea* and *Melastoma* was 0.50 or 0.51, indicating the two more old WGD events of both genera were shared by their ancestor, and their slight difference might be attributed to their differential substitution rates.

### Identifications of the transcription factors related to the anthocyanin biosynthesis

**Figure 3:**
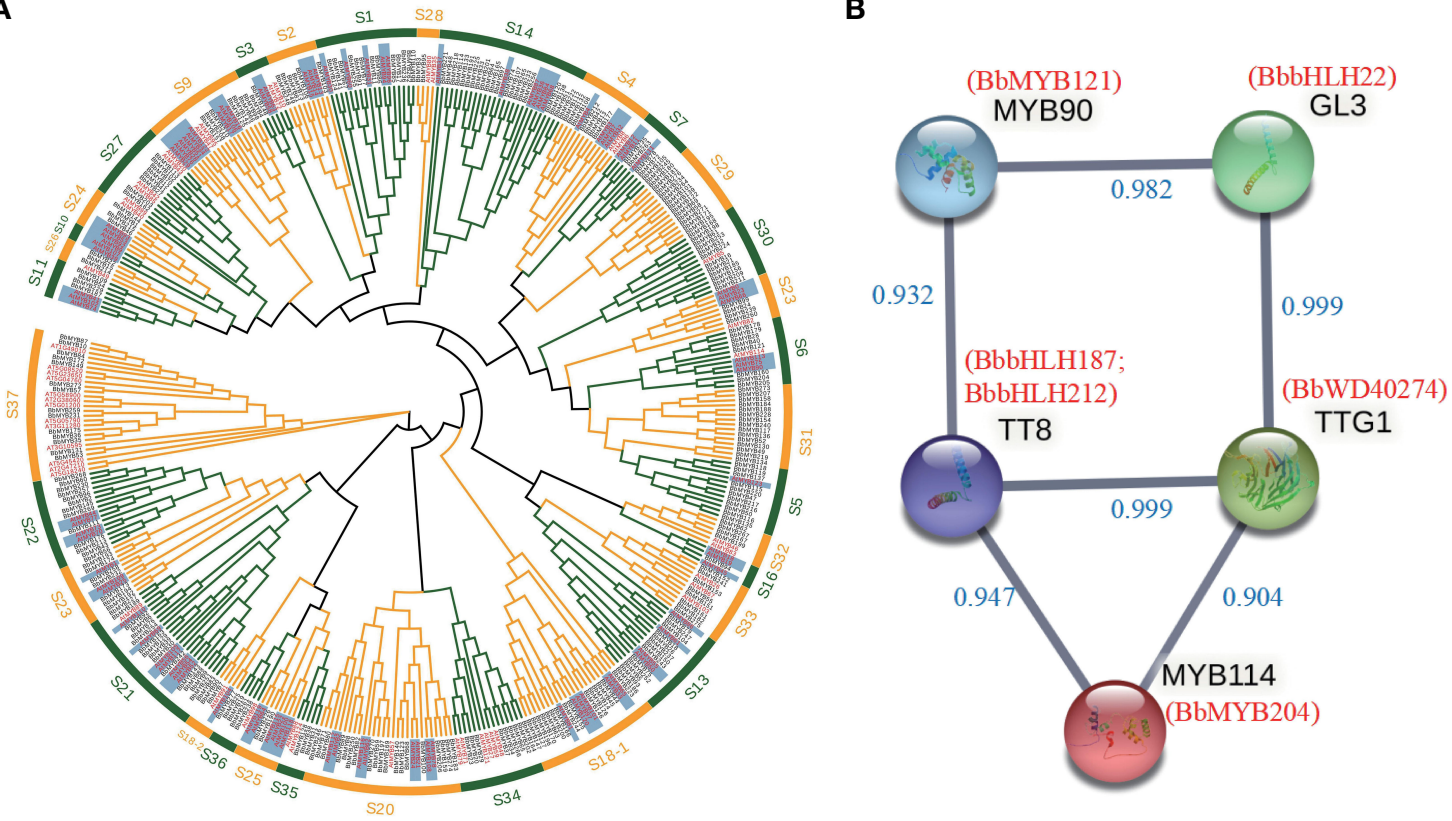
Identification of transcription factors involved in anthocyanin biosynthesis in *Bathea bathei*. **(A)** Phylogenetic analysis of the R2R3-MYB gene families of *B. barthei* and Arabidopsis thaliana. The tree was constructed using the maximum likelihood method with 1000 bootstrap replicates. The R2R3-MYB subfamilies are indicated by different colors; **(B)** Protein-protein interaction network of candidate transcription factors MYBs, bHLHs and WD40s involved in anthocyanin biosynthesis in *B. barthei*. The network was constructed using STRING v11.5 with a minimum required interaction score ≥0.9. Nodes represent candidate genes, and edges represent predicted interactions.

We identified 489 *MYB*, 222 *bHLH*, and 330 *WD40* transcription factor gene families in the *B. barthei* genome (**Supplementary Tables S14**–**S16**). Phylogenetic analysis classified these into subfamilies, providing clues to their functions (**Figure 3A**; **Supplementary Figures S10**, **S11**). For instance, the R2R3-MYB subfamily containing *AtMYB75*, *AtMYB90*, *AtMYB113*, and *AtMYB114* essential for anthocyanins in Arabidopsis fell into group 6 (**Figure 3A**). Within this subfamily, eight *B. barthei* transcription factors were identified, including *BbMYB28*, *BbMYB40*, *BbMYB121*, and *BbMYB204*. Four key bHLH anthocyanin regulators in Arabidopsis - *TT*8, *EGL3*, *GL3*, and *MYC1* - also had sixteen ortholog members in subgroups IIIf and IX in *B. barthei* (**Supplementary Figure S10**). Additionally, three members homologous to model plant anthocyanin regulators belonging to WD40 groups also were also identified in the genome assembly of *B. barthei* (**Supplementary Figure S11**). Overall, more orthologs in *B. barthei* for the three transcription families than in Arabidopsis.

RNA-seq identified 3699 upregulated and 4053 downregulated differentially expressed transcripts (DETs) between white and red-purple ecotypes. Among these DETs, there were 184 MYBs, 156 bHLH, and 304 WD40s. Enrichment analysis revealed that many of the DETs were related to flavonoid/anthocyanin biosynthesis (**Supplementary Table S17**). Of these DETs, 9 TFs were associated with anthocyanins (**Supplementary Table S18**).

Protein interaction networks identified 6 DETs as high-confidence nodes, including 4 MBW complex regulators (*BbMYB204* homologous to *MYB114*, *BbbHLH187*, *BbbHLH212* homologous to *TT8*, *BbWD40_274* homologous to *TTG1* in Arabidopsis) key for anthocyanin biosynthesis (**Figure 3B**).

### Population structure and demographic history of *B. barthei*

**Figure 4:**
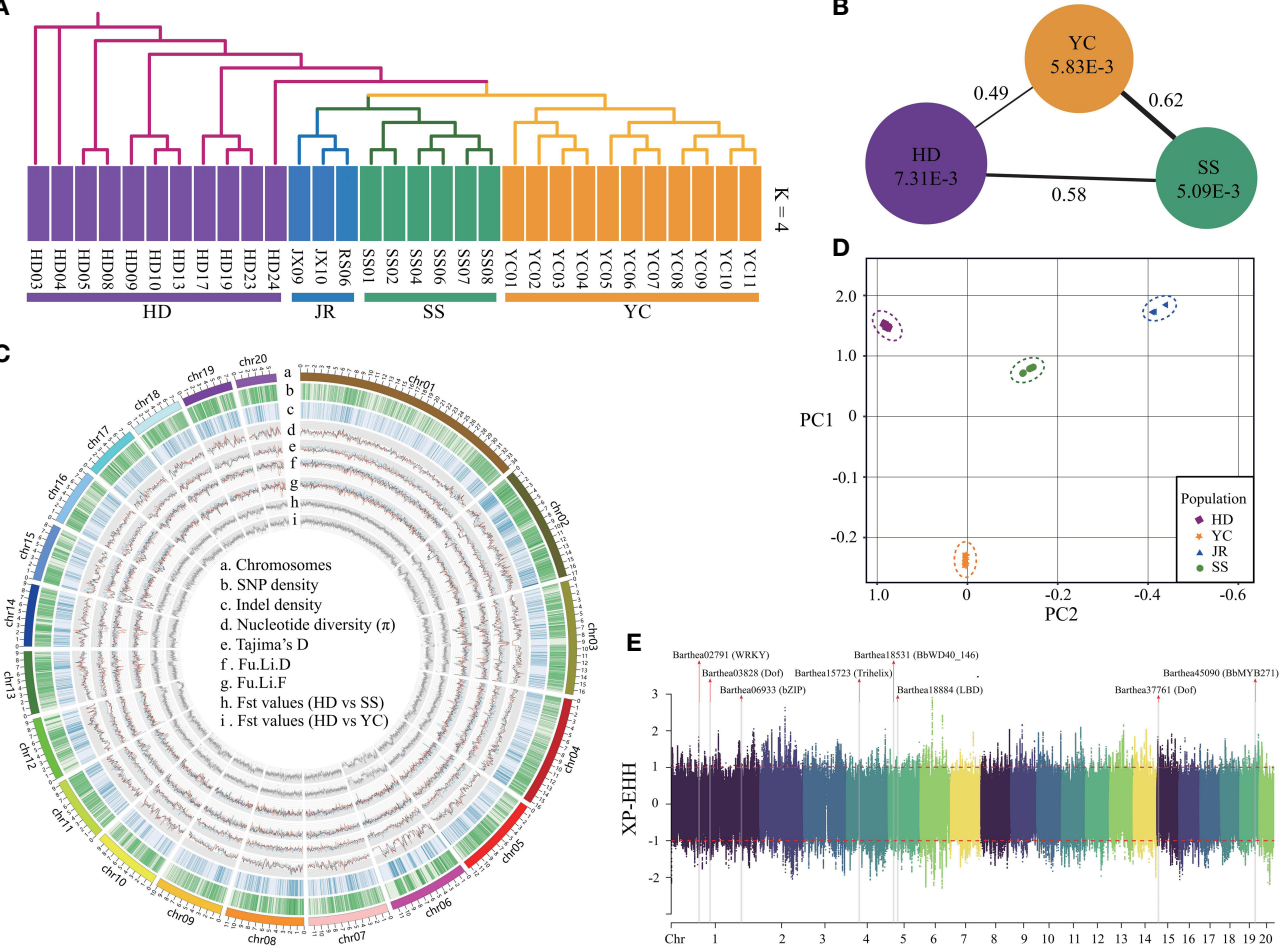
Population genetic components and genome wide natural selection signal scan for different geographic populations of *Barthea barthei*. **(A)** Population structure analysis using admixture and phylogenetic methods; **(B)** The overall polymorphism and genetic differential index between different population; **(C)** Circos plot for the genome wide polymorphisms and genetic differential index across twenty pseudochromosomes of geographic populations of *B. barthei*; **(D)** PCA plots of the first two components; **(E)** Manhattan plot for the genome wide XP-EHH score distributions across twenty pseudochromosomes between HD and YC population with a sliding window size of 20 kb and a step size of 2 kb.

Whole genome resequencing of 31 *B. barthei* accessions from five representative populations (**Supplementary Figure S12C**; **Supplementary Table S1**) generated ~700Gb of reads with 22.1× average depth and 81.41% mapping rate (**Supplementary Table S19**). Strict filtration identified 14,936,541 SNPs and 2,202,590 small indels (<10 bp), with 8.57% of SNPs located in exons and 11.61% in introns (**Figure 4C**; **Supplementary Tables S20**, **S21**). Admixture analysis revealed that k = 4 was the optimal number of populations, with the lowest cross-validation error (**Supplementary Figure S12A**). The four groups corresponding to HD, SS, YC and JR were clearly separated (**Figure 4A**; **Supplementary Figure S12B**). With k=2, YC and SS populations were distinct from the remaining populations. Principal component analysis (PCA) explained 27.21% and 24.35% of the variance along PC1 and PC2, respectively, separating four geographic populations (**Figure 4D**). Consistent with the admixture and PCA analyses, neighbor-joining (NJ) phylogenetic treeing showed the 31 *B. barthei* accessions clustering into four major lineages (**Figure 4A**). Genome-wide diversity estimated using π and Tajima’s D revealed the SS population (mean π =5.09x10^-3^) had lower genetic diversity than the YC and HD populations (mean π = 5.83x10^-3^ and 7.31x10^-3^, respectively) (**Figure 4B**). Tajima’s D values for the populations (D_SS_ = 0.373, D_YC_ = 0.003, D_HD_ = 0.160) indicated *B. barthei* likely experienced balancing selection or past population contractions. F_ST_ values between the populations ranged from 0.49 to 0.62, with higher differentiation between SS and the others (**Figure 4B**; **Supplementary Figure S13**; **Supplementary Table S22**).

**Figure 5:**
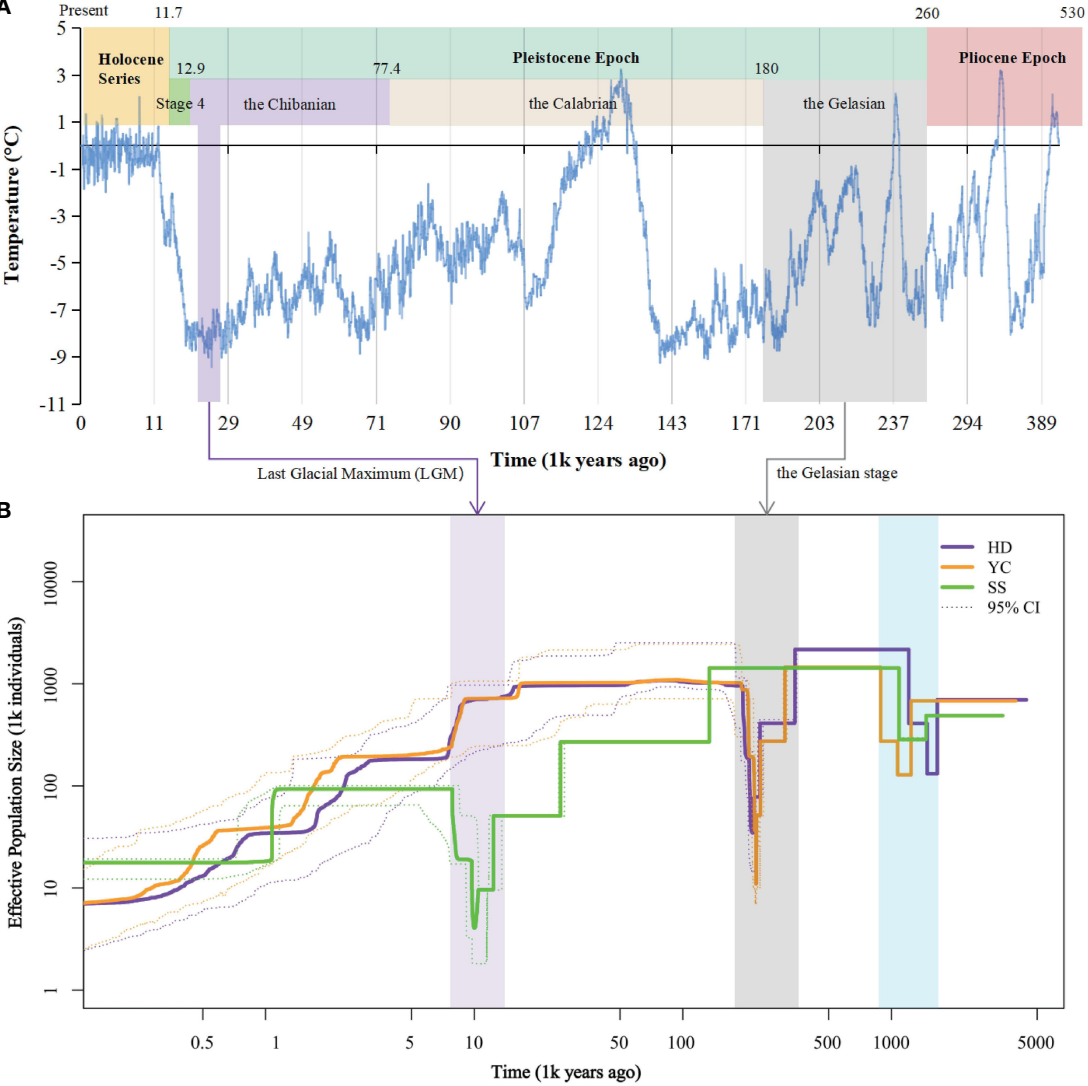
Demographic history of *Barthea barthei* populations. **(A)** Specific presentation with ice core data for the past 420,000 years (Petit et al., 1999). **(B)** Historical effective population size for HD, YC and SS population. Stairway plot showing that each population underwent two bottlenecks respectively. The first bottleneck is shared by the three populations. Both YC and HD underwent a second bottleneck in the Gelasian stage, and the SS population experienced a second population bottleneck in the LGM epoch. Abbreviations for populations as following: HD, Huidong population in Guangdong province; YC, Yanchun population in Guangdong province; SS, Shangsi population in Guangxi Province.

Inference of historical effective population size (Ne) revealed two bottleneck events for the SS, YC, and HD populations (**Figure 5**). The first occurred between 148.4-107.1 million years ago (Mya) in all populations. YC and HD underwent a second bottleneck during the Gelasian (22.5-21.6 Mya) before recovering, followed by gradual Ne declines in the Last Glacial Maximum (LGM). In contrast, SS maintained a relatively constant Ne until the Eemian Interglacial when it started declining gently. A second bottleneck then occurred for SS during the LGM (2.9-1.9 Mya). The timing of the second bottlenecks coincided with known cold climatic periods. Notably, after the first bottleneck, SS showed a divergent evolutionary history from YC and HD, likely affected by its higher differentiation.

### Genomic signals of natural selection

To minimize the effects of geographic isolation, we examined genomic signals of petal color variation between proximate HD and YC populations. Based on the shared regions with the top 5% of F_ST_ (F_ST_ ≥0.74), and top and bottom 5% of θ_π_ ratio (log2) (θ_π_≥ 0.30, θ_π_ ≤1.49), 2133 regions (10.85 Mb, 4.62% of the assembled genome) under selection were identified, consisting of a total of 1580 candidate positively selected genes (**Supplementary Figure S14**). Based on top and bottom 5% of XP-EHH scores, 1168 normalized genomics regions were identified, containing 4880 genes (**Figure 4E**). By cross validation among the three approaches, 194 high confident selected genes were determined (**Supplementary Table S23**). Four GO terms were significantly enriched for these positively selected genes (**Supplementary Table S24**). The gene ontology term ‘root development’ (GO0048364, FDR=0.008) provides clues about adaptation to the distinct soil types. The purple/red ecotype occupied high mountaintops with barren, rocky soils lacking in nutrients. By contrast, the white ecotype occurred in the more hospitable understory soils, which were relatively fertile loamy earths with higher organic content. For these conserved high confident genes set, a total of five transcription factors were identified, including *WRKY28*, *Dof*, *bZIP*, *Trihelix*, *LBD* (**Supplementary Table S25**; **Figure 4E**).In the positively selected gene sets identified by F_ST_ and θ_π_ ratio. There were two GO terms associated with anthocyanin biosynthesis, including regulation of anthocyanin biosynthetic process (GO0031540; P: 1.8E-02) and anthocyanidin 3-O-glucosyltransferase activity (GO0047213; P: 4.7E-02) (**Supplementary Table S26**). Two positively selected genes *Barthea19645* and *Barthea26008* were identified to be differentially expressed between purple and white ecotypes respectively (Padj < 0.05; log|FC| > 1), in which *Barthea19645* was a homologue with *AtMYB113* (*AT1G66370.1*) and *Barthea26008* was a homologue with *UGT78D2* (AT5G17050.1) (**Supplementary Table S27**).

## Discussion

### Ecological significance of flower color shifts in *B. barthei*

Anthocyanins are essential for generating the beautiful palette of plant colors in nature. The anthocyanin pigments are responsible for the varied coloration across many plant species. By accumulating in the vacuoles of plant cells, especially in flowers and fruits, anthocyanins can exhibit a wide spectrum of hues. The specific types and combinations of anthocyanin compounds, along with cell structure and pH, determine the distinct pink to purple shades observed in plants (Zhao and Tao, 2015). The production of anthocyanin pigments is controlled by MBW protein complex. These three transcription factor groups collectively regulate anthocyanin biosynthesis across a wide variety of plant species (Spelt et al., 2002; Ramsay and Glover, 2005; Hichri et al., 2011). In this study, the red-purple ecotype is commonly found occupying high-elevation, open habitats. In contrast, the white ecotype inhabits low-elevation, forest understory environments.

The distinct flower colors observed between the red-purple and white ecotypes of *B. barthei* likely represent adaptations to the contrasting light regimes in their habitats. The red-purple pigmentation present at high elevations may protect reproductive tissues against intense UV radiation and drastic temperature fluctuations. Anthocyanins can act as UV-absorbing sunscreens to prevent damage to cells when sunlight exposure is high (Gould et al., 2002). In contrast, the lack of red pigments in the low elevation flowers could enable more efficient light capture in the shaded understory environment. Rather than attracting pollinators, the divergence in flower color may reflect adaptations to the gradients in sunlight, moisture, and temperature across the species’ elevation range. Further examination of additional functional traits related to stress tolerance is warranted. Tracking performance differences under UV radiation and temperature extremes could clarify the adaptive benefit of anthocyanins. The production of flavonoids like anthocyanins is often induced by light (Jaakola, 2013), suggesting their accumulation may correlate directly with sunlight levels. Overall, this system provides an opportunity to explore the genetic changes enabling ecological adaptation to the distinct abiotic environments occupied by the *B. barthei* ecotypes.

### Genomic and population insights into mechanisms of flower color adaptations in *B. barthei*

Whole genome duplications were essential to the evolution of traits innovations in plants (Van de Peer et al., 2017). In this study, two recent tetraploidizations specific to Melastomaceae were identified in the genome of *B. barthei.* These tetraploidizations resulted in massive duplicated gene content, with over 70% of genes retained in collinear blocks. This is more than 10 times the proportion in the closely related *Eucalyptus*, which has only 2340 collinear genes (approximately 6.44%) and underwent an early lineage-specific paleotetraploidy event around 109.9 million years ago (Myburg et al., 2014). Moreover, the percentage of retained genes was twice as high as in Arabidopsis (23.1%), which experienced two additional WGDs (designated as α and β) beyond the shared eudicot-wide triplication event (γ) (Paterson et al., 2004; Barker et al., 2009; Jiao et al., 2011). The extraordinary proportion of collinear genes in *B. barthei* suggests that multiple WGDs, particularly a very recent one, have significantly influenced its highly collinear genome structure. A notable consequence of these recent WGD events in *B. barthei* was the expansion of gene families and the rapid evolution related to anthocyanin biosynthesis, as shown by the core regulators of MBW complex. Additionally, four transcription factors of the MBW complex that were differentially expressed provides insights into the divergence of flower color between the two ecotypes. Prior studies indicates that *MYB114* (homologous to *BbMYB204*) upregulates anthocyanins in conjunction with *TTG1* (homologous to *BbWD40_274*) and *TT8* (homologous to *BbbHLH187*, *BbbHLH212)*. while *TTG1*-containing complexes control the expression of anthocyanin-related genes such as *DFR* and *BAN* (Gonzalez et al., 2008). *TTG1* also enhances anthocyanin production by facilitating MYB-bHLH interactions with the MBW complex (Ramsay and Glover, 2005; Xu et al., 2015; Airoldi et al., 2019). Furthermore, *TT8* acts via at least 6 redundant MBW complexes, serving as a key regulator of both anthocyanin and proanthocyanidins (Xu et al., 2013). The examination of population structure and demography uncovered two historical bottlenecks, which substantially reduced effective population size. These contractions may have decreased genetic diversity but concurrently facilitated the rapid rise in frequency of new beneficial alleles (Excoffier et al., 2009; Turner et al., 2010). Within the set of genes under strong positive selection, only the *Barthea19645* homologue to *AtMYB113* and the *Barthea26008* homologue to *UGT78D2* have been directly associated with anthocyanin production. In *Arabidopsis thaliana*, overexpression of *AtMYB113* leads to marked increases in pigment production, with the MBW complex of *AtMYB113* predominantly regulating late-stage genes in the phenylpropanoid pathway (Gonzalez et al., 2008). UGT78D2 is an enzyme that glucosylates the 3-position of the flavonoid C-ring, coding for an anthocyanidin 3-O-glucosyltransferase, which influences anthocyanin accumulation in the plant tissues (Kubo et al., 2007; Kim et al., 2012). These offer candidate targets of adaptive evolution contributing to flower color differentiation between ecotypes.

By utilizing approaches that include the dynamics of gene family evolution, comparisons of transcriptome profiles, and population genomics, we aimed to fully unravel the underlying mechanism of flower color divergences between the two *B. barthei* ecotypes. However, the results obtained from different methodologies were inconsistent, and some of the identified genes were not relevant to flower color. The sources of such inconsistency could be diverse, including varying sources of genetic, the entanglement of other divergent traits with flower color, and complex population history. For instance, expansions and evolution within gene family captured interspecies divergence rather than intraspecies variation observed in the flower color differences between the two ecotypes. Additionally, certain genes that were identified were not inclusively related with flower color. For instance, the five positively selected transcription factors, namely *WRKY28* (Khoso et al., 2022), *Dof* (Zou and Sun, 2023), *bZIP* (Dröge-Laser et al., 2018), *Trihelix* (Kaplan-Levy et al., 2012), *LBD* (Rubin et al., 2009) did not have a direct association with flower color. These transcription factor families instead play crucial roles in the regulation of gene expression, coordinating various aspects of plant growth and development, and in the plant’s response to a wide array of biotic and abiotic stresses.

In this study, although our stringent approaches may have overlooked some important genes, these multi-faceted genomic evidence supports the notion that two recent tetraploidizations provided raw genetic material. Past demographic shifts likely enabled selective sweeps, and divergent selection was instrument in driving allele frequency changes at trait-associated loci. Collectively, these evolutionary processes likely facilitated the emergence of variants that alter the regulation of the anthocyanin pathway, which may have facilitated the divergence in floral pigmentation as *B. barthei* adapted to the contrasting environments of the two ecotypes.

## Conclusion

In this study, we provide a high-quality genome assembly and annotations for the monogenic species *B. barthei*, representing the first genome resource for this genus. Comparative genomics and transcriptomics analyses of *B. barthei*, a species with natural variation in flower color, revealed rapid expansions and positive selection of anthocyanin-related transcription factors. These findings, coupled with the identification of two recent whole-genome duplications, suggest that the evolution of flower color divergence in *B. barthei* was driven by a complex interplay of genetic and evolutionary forces. The rapid expansions of anthocyanin-related gene families provided the raw genetic material for the evolution of novel regulatory interactions and the diversification of anthocyanin pigmentation. Positive selection acted on these expanded gene families, driving the divergence of flower color between the red-purple and white ecotypes. The two whole-genome duplications further facilitated adaptive evolution by providing additional copies of genes that could be modified by positive selection. Our study highlights the importance of considering the evolutionary history of a species, including polyploidy events, when studying the genetic basis of adaptation. The combination of rapid expansions, positive selection, and whole-genome duplications suggests that the evolution of flower color in *B. barthei* was a complex process involving multiple genetic and evolutionary mechanisms.

This study provides a comprehensive genomic and evolutionary framework for understanding the genetic basis of flower color divergence in *B. barthei* and contributes to our understanding of the evolutionary mechanisms underlying ecological adaptation in plants. The identification of candidate genes under positive selection provides a starting point for future functional studies to elucidate the molecular mechanisms underlying flower color divergence in *B. barthei* and to investigate the role of polyploidy in adaptive evolution. Indeed, as evidenced in genomic scan for signature of natural selection, for the high confident gene sets under selection, some genes associated with development and growth, and response to biotic or abiotic stresses enlighten us that many other import ecological factors have driven the divergence between the purple/read ecotype and white ecotype. Further research is needed to investigate the specific ecological factors that have contributed to this divergence.

## Data availability statement

The raw data of whole genome sequencing and RNA sequencing are deposited in the Genome Sequence Archive (GSA accession: CRA012896 under the project PRJCA020264) in China National Genomics Data Center (NGDC) database. The genome assemblies and annotations were available with accession: GWHDUDN00000000.

## Author contributions

WH: Data curation, Formal analysis, Investigation, Methodology, Software, Validation, Visualization, Writing – original draft, Writing – review & editing. BX: Data curation, Formal analysis, Writing – original draft, Writing – review & editing, Investigation, Methodology, Software. WG: Investigation, Methodology, Writing – review & editing. ZH: Formal analysis, Software, Writing – original draft. YL: Conceptualization, Investigation, Methodology, Project administration, Resources, Supervision, Writing – review & editing. WW: Conceptualization, Formal analysis, Funding acquisition, Investigation, Methodology, Project administration, Software, Supervision, Validation, Writing – original draft, Writing – review & editing.
