# Supplementary material for: De novo genome assembly and population genomics of a shrub tree Barthea barthei (Hance) krass provide insights into the adaptive color variations
Source: Front Plant Sci. 2024 May 1;15:1365686. doi: 10.3389/fpls.2024.1365686 (PMC11094225; doi:10.3389/fpls.2024.1365686)
Supplement: Supplementary file 1 [file DataSheet_1.zip › Figure S1-S14.DOCX]

**Supplementary Figures**

**
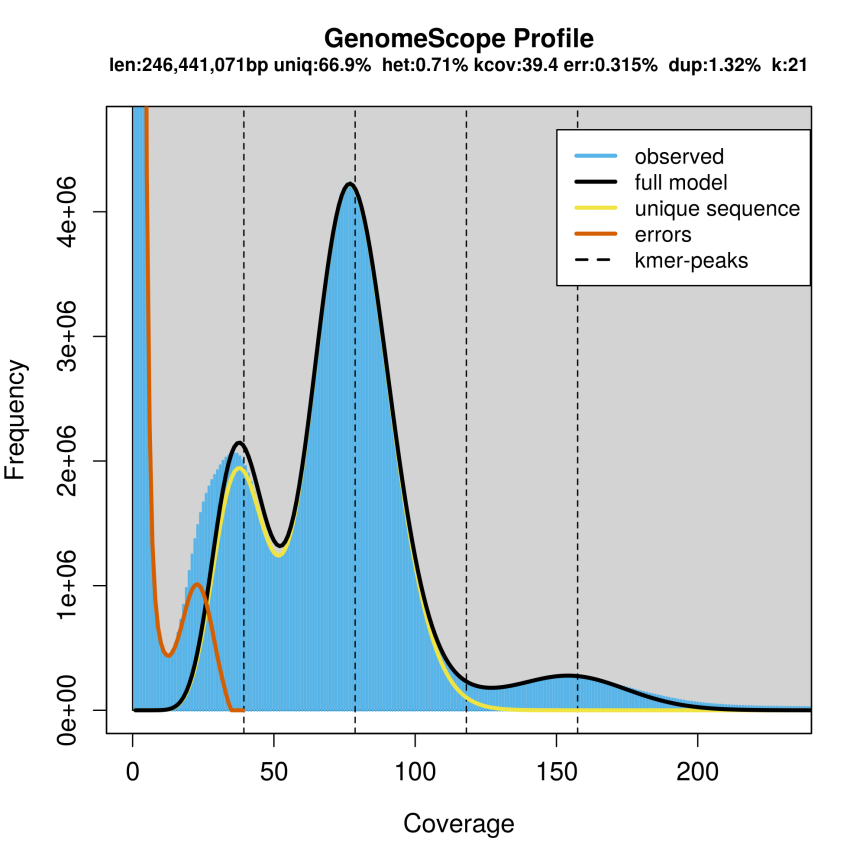
**

**Figure S1.** Genome survey of *Barthea barthei* using 21-mer analysis. X axis shows k-mer coverage and Y axis shows k-mer frequency. The genome size was measured to be 246 Mb.


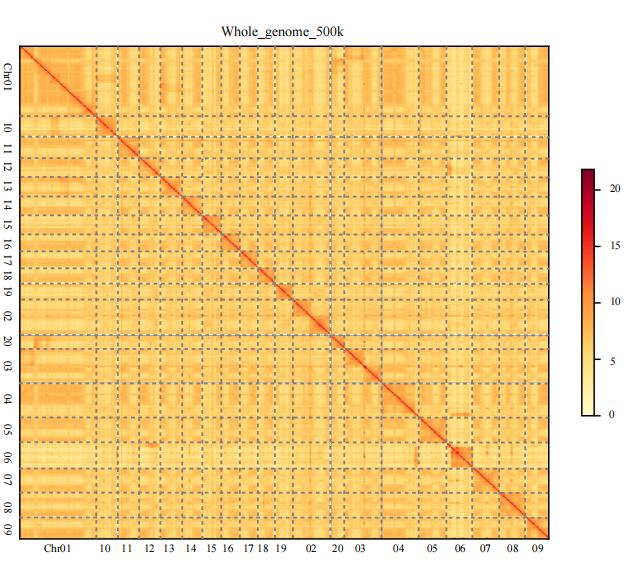


**Figure S2.** Hi-C heatmap of the *Barthea barthei* genome demonstrating genome-wide all-by-all interactions. The interaction signal strength around the diagonal was stronger, 01-20 representing the 20 pseudochromosomes.


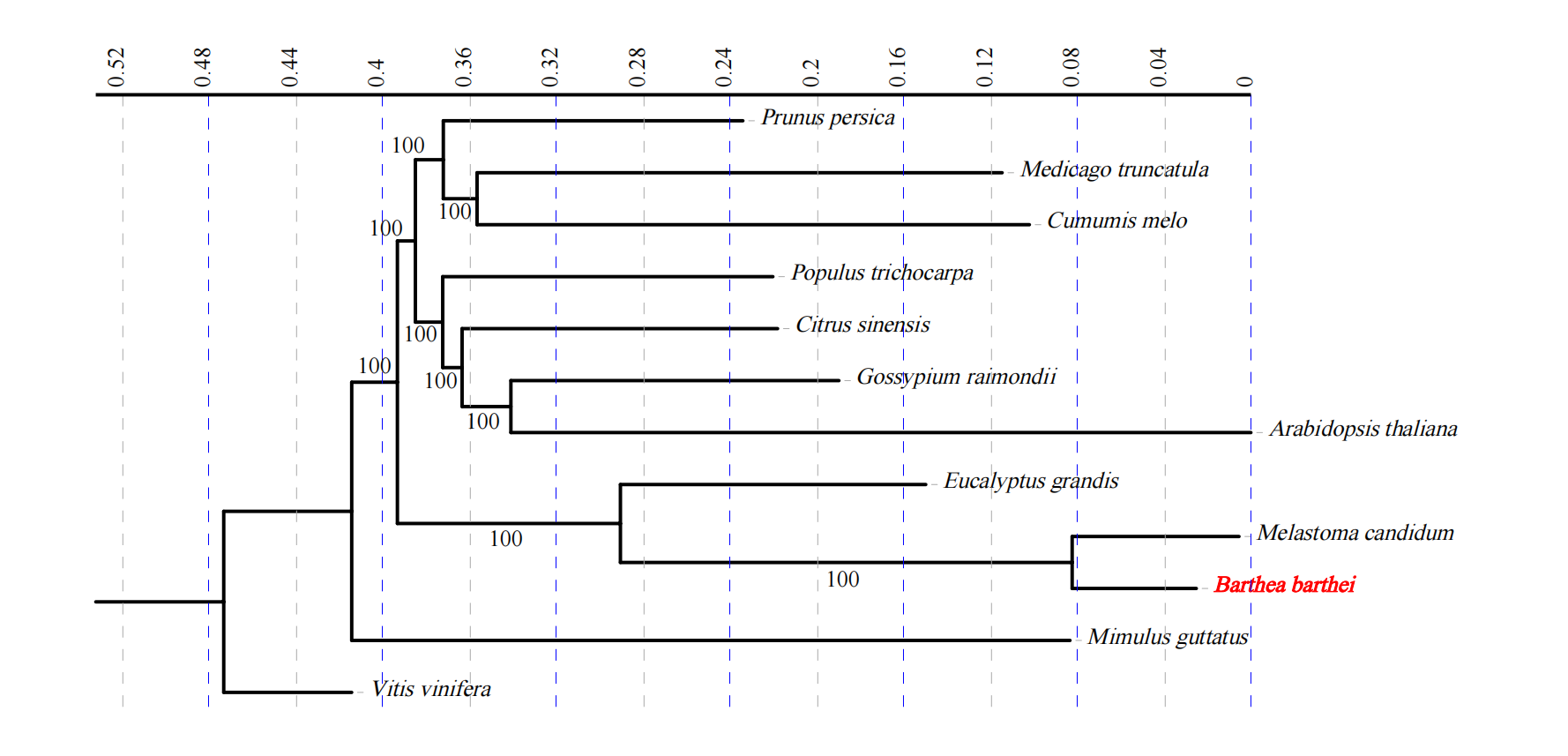


**Figure S3.** The phylogenetic trees of *Barthea barthei* and other related species base on concatenated single copy genes for 12 plant species**.** Numbers near the nodes are support rates for 1000 bootstraps; Number above the branches are branch lengths.

**
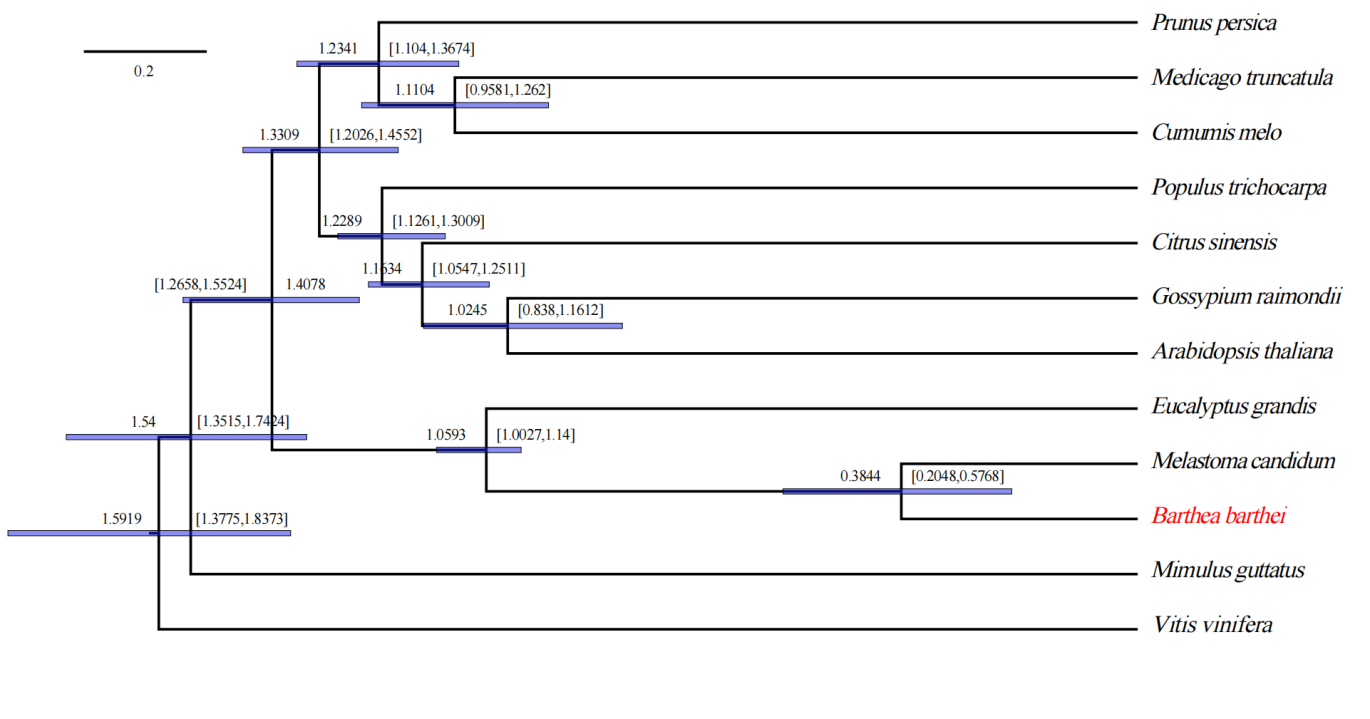
**

**Figure S4.** Phylogenetic tree shown for the divergence time among *Barthea barthei* and other related species, mean divergence time and confidence.

**
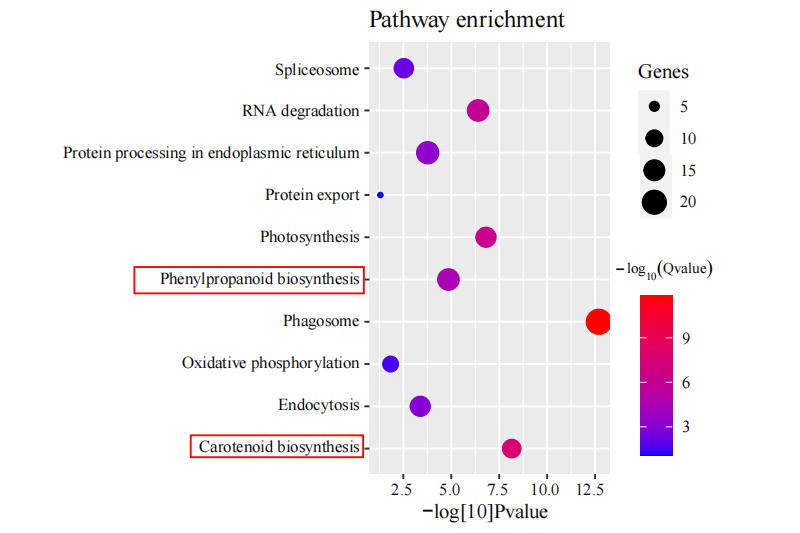
**

**Figure S5.** KEGG enrichment of significantly expanded gene families in *Barthea barthei* using hypergeometric test/Fisher's exact test.

**Figure S6.** Gene duplication types of *Barthea barthei* genome**.** The distribution of the five duplication types classified by MCScanX as follows: Singleton: no duplication; WGD/segmental: whole genome or segmental duplications (collinear genes in collinear blocks); Tandem: consecutive duplication; Proximal: duplications in nearby chromosomal region but not adjacent; Dispersed: duplications of modes other than tandem, proximal or WGD/segmental.


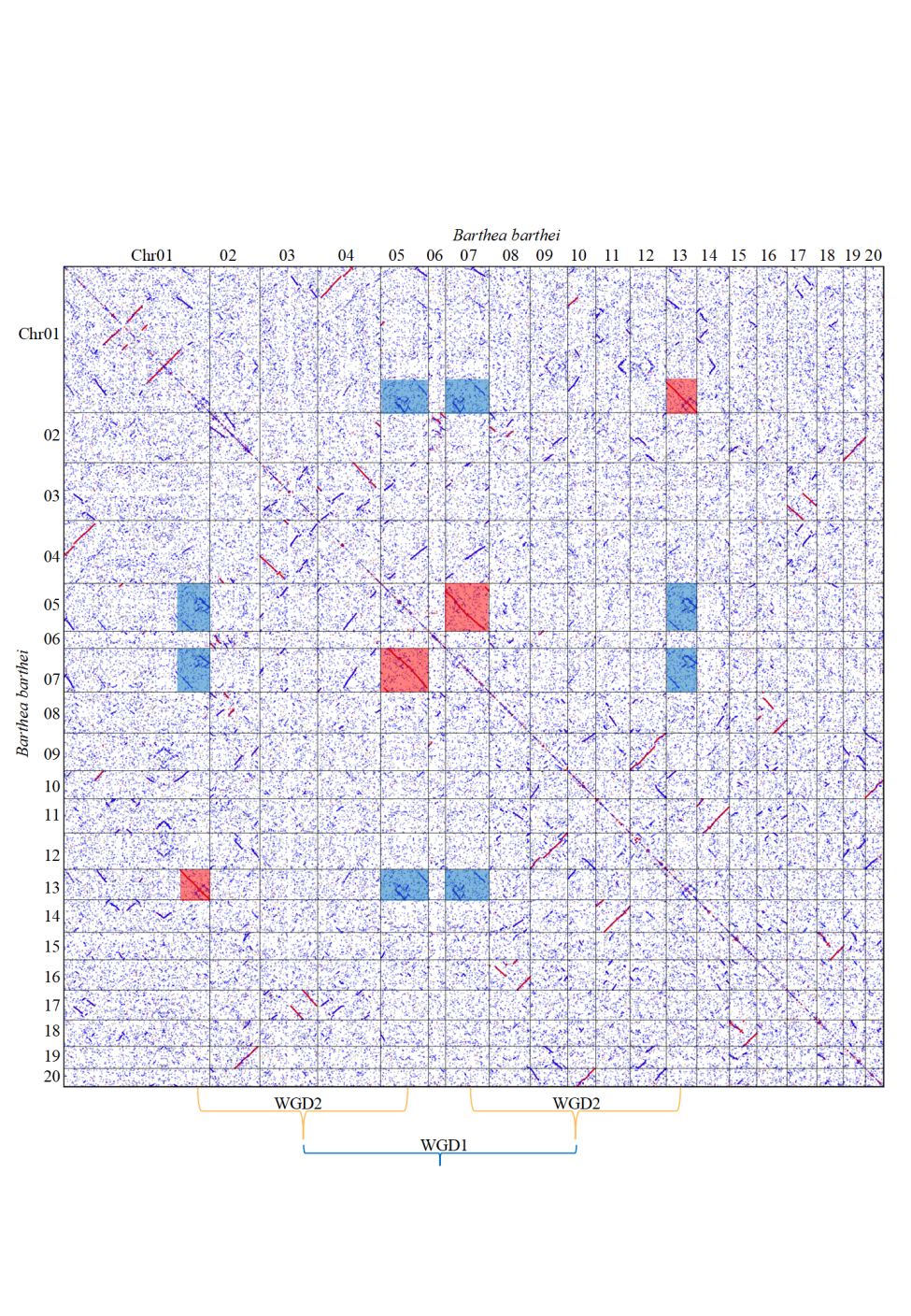


**Figure S7.** Dotplot of homologous genes in *Barthea barthei*. The red dots show the best matched genes, blue dots secondarily matched ones, and the gray ones being produced by more ancient or dispersal duplicated genes.


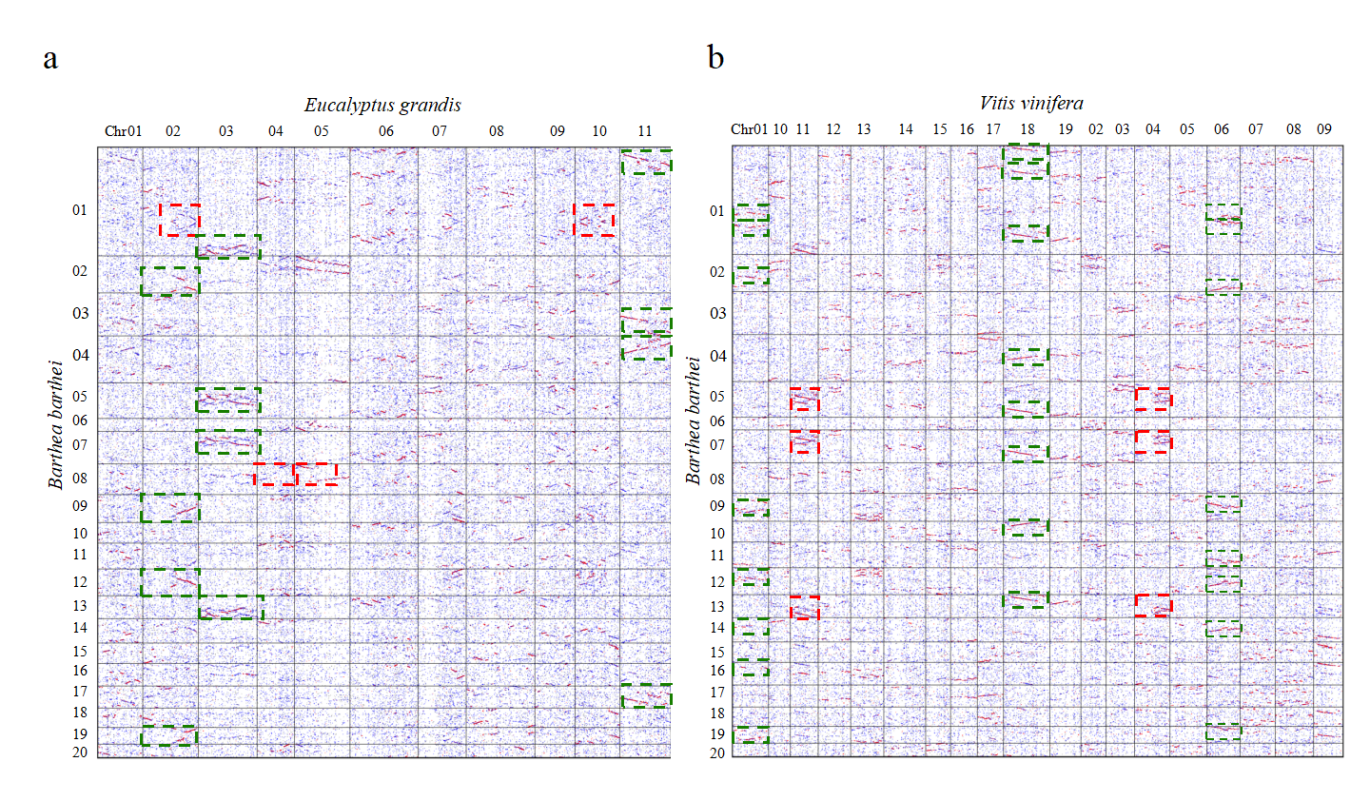


**Figure S8.** Orthologous gene pairs in syntenic blocks between *Barhea barthei* and related species. Dots represent the position of orthologous gene pairs. **a** syntenic gene pairs between *B. barthei* and Eucalyptus; **b** syntenic gene pairs between *B. barthei* and grape.


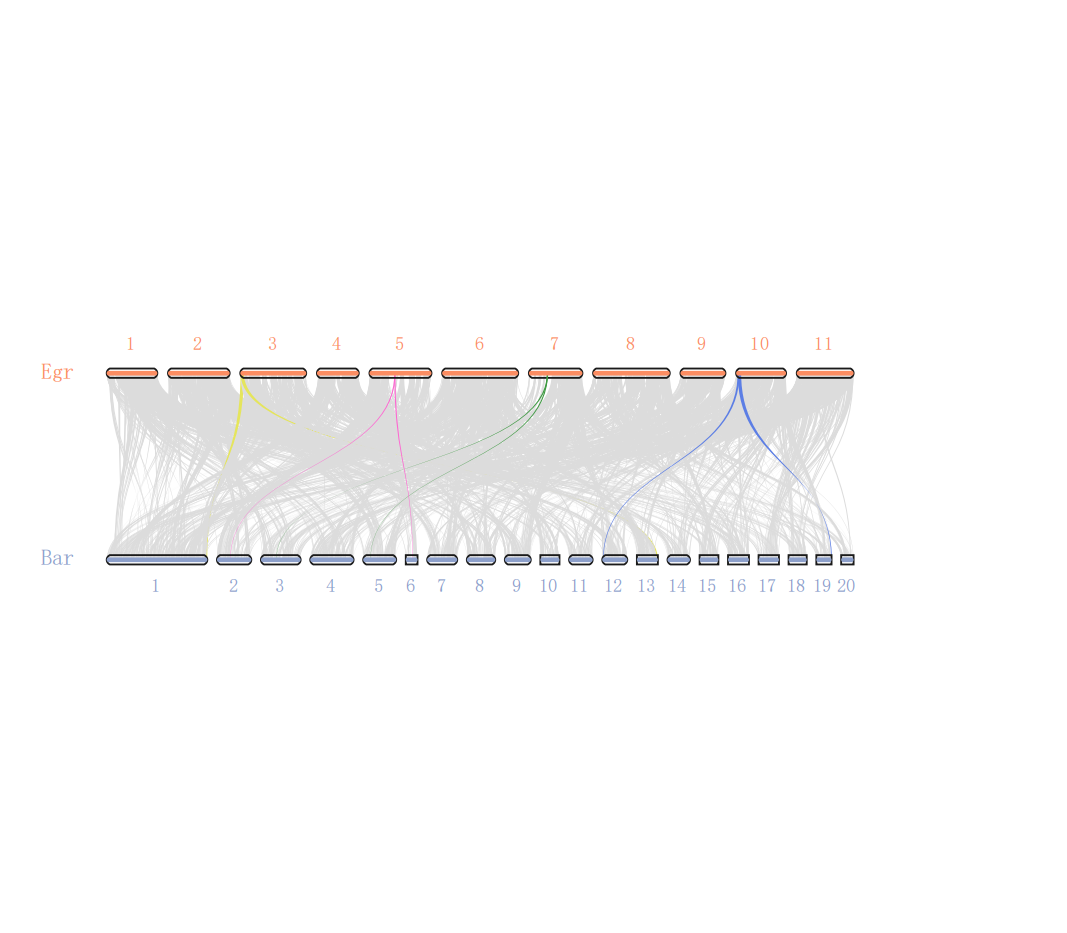


**Figure S9.** Macrosyneny between *Barthea barthei* and Eucalyptus**.** Microsynteny regions was highlighted to indicate the 1:2 syntenic depth between *B. barthei* and Eucalyptus.


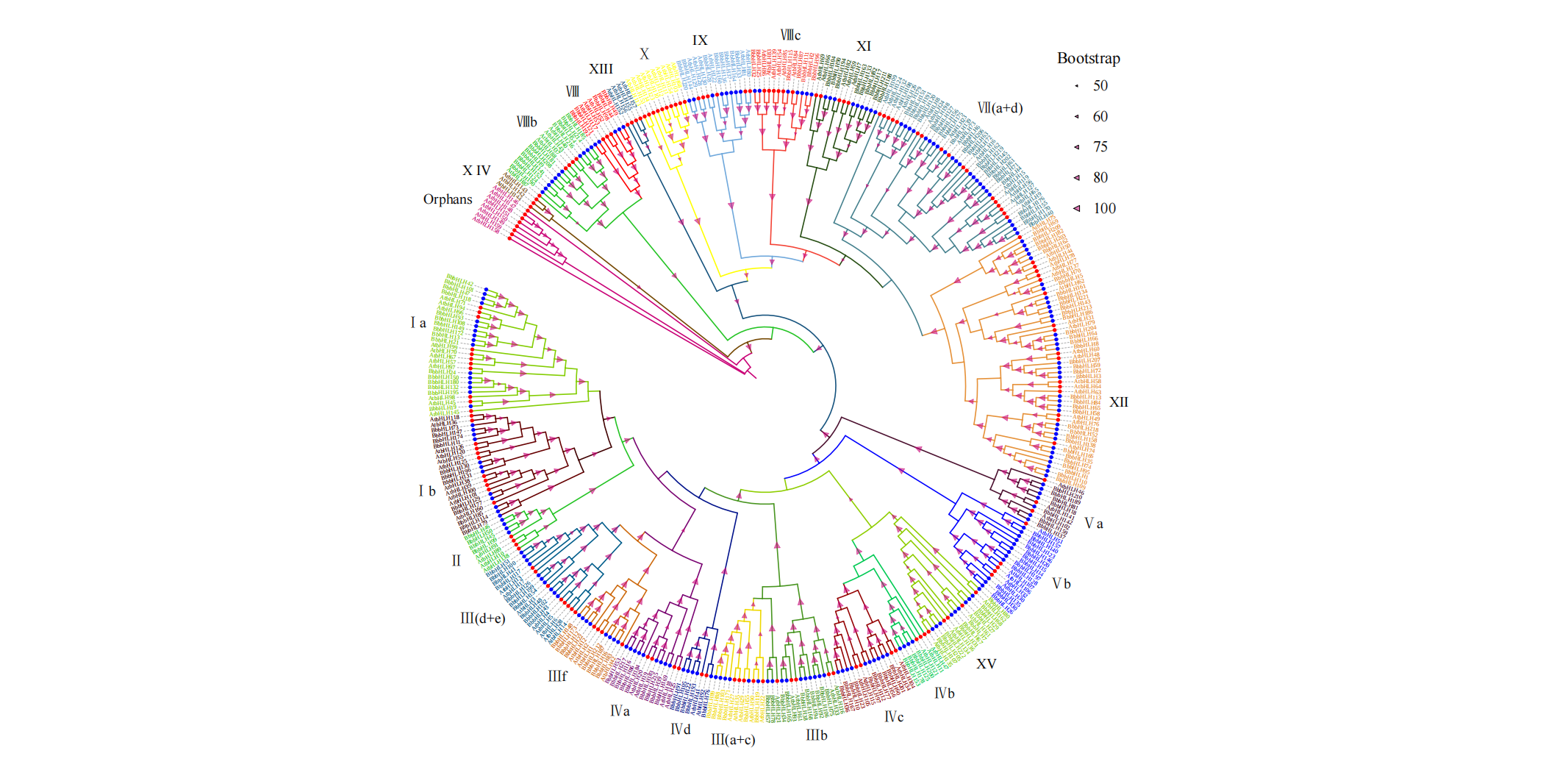


**Figure S10.** Phylogenetic tree of bHLH gene families between *Bathea barthei* and *A. thaliana*.


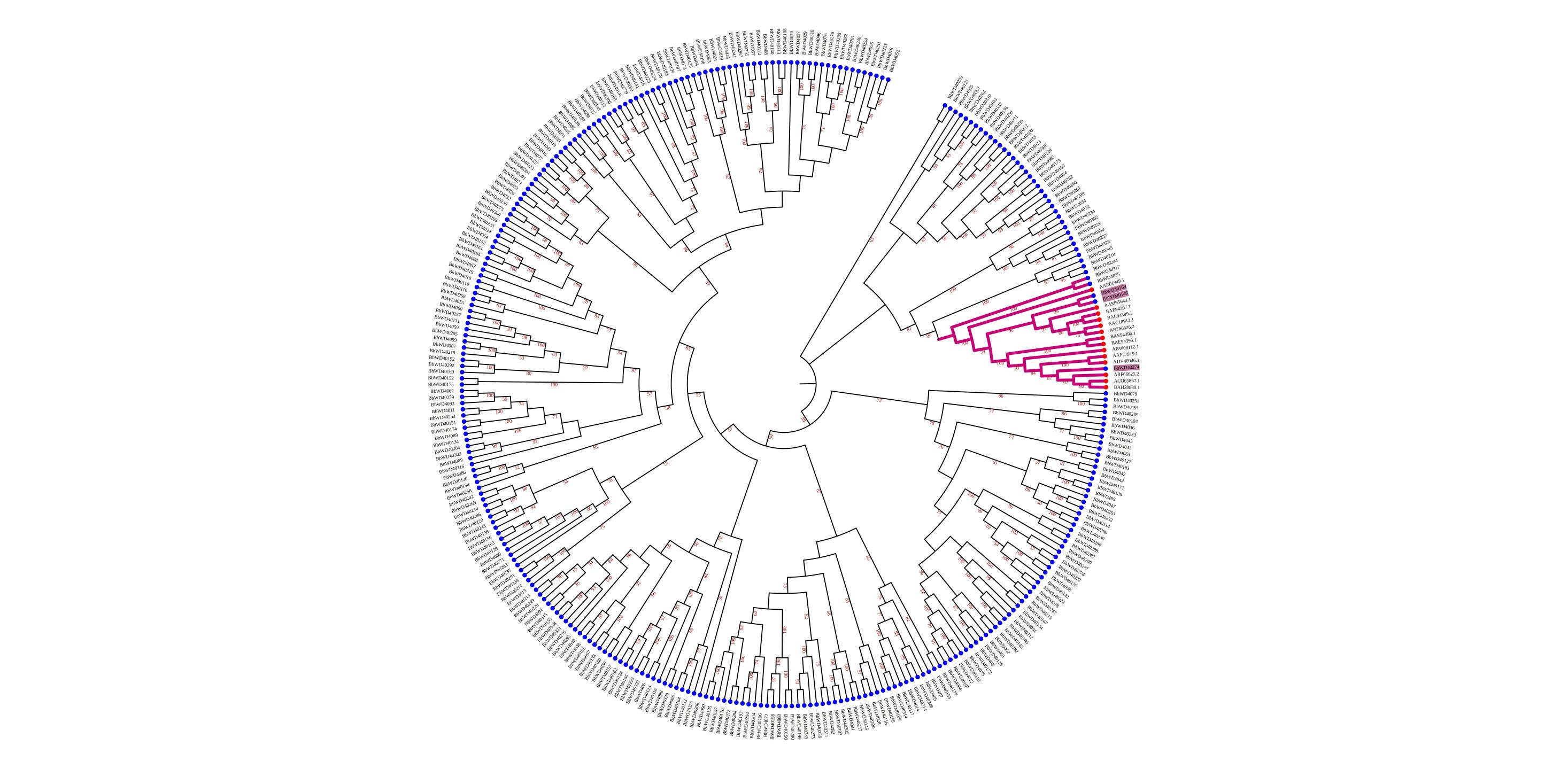


**Figure S11.** Phylogenetic tree of WD40 gene families of *Bathea barthei*.

**
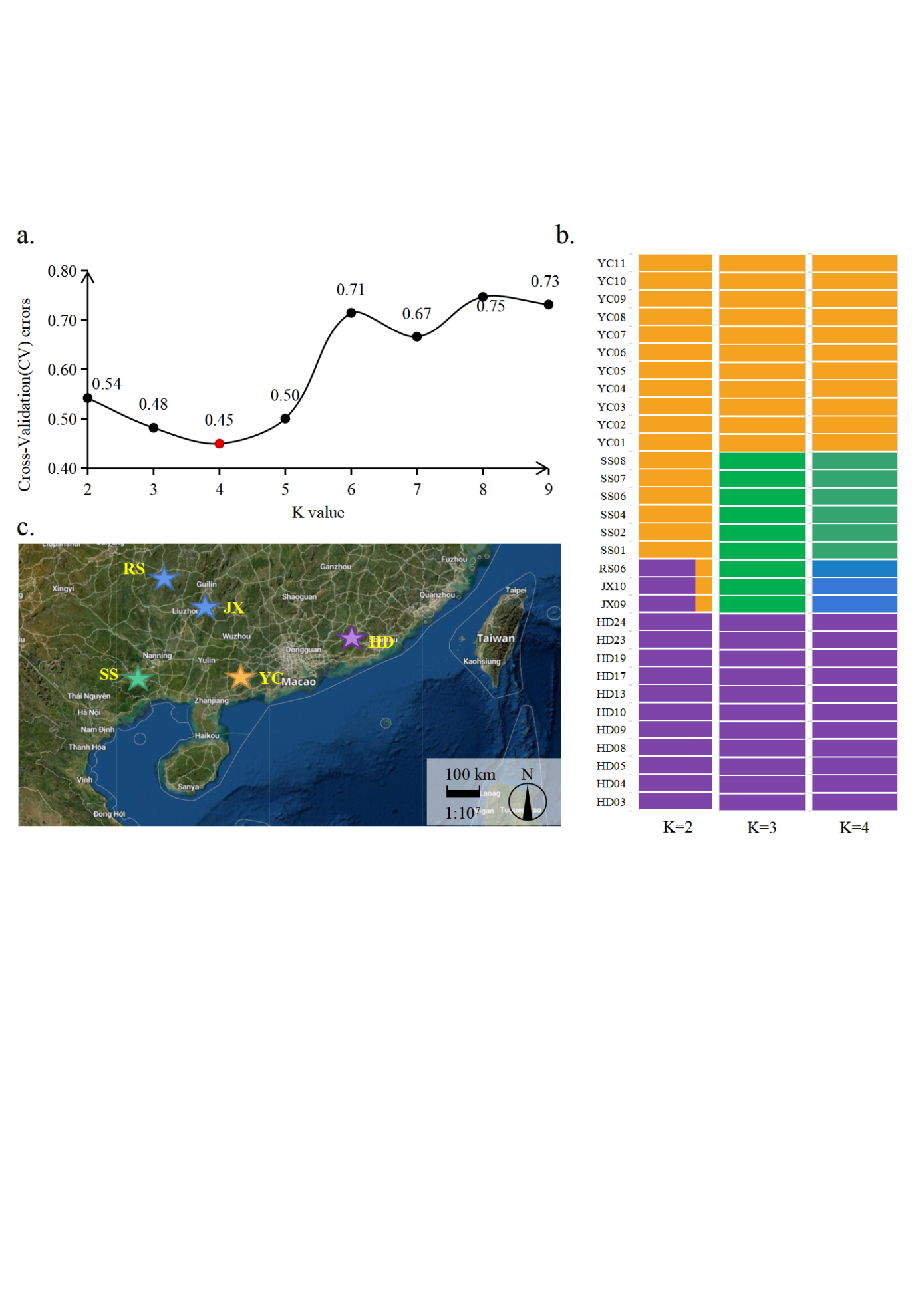
**

**Figure S12.** Maximum likelihood estimation of the genetic components for the population of *Barthea barthei* implemented in STRUCTE. a.Corss-validation errors across different K valules; b. The genetic components with different K value; c. Sampling location map for *B. barthei* in this study.


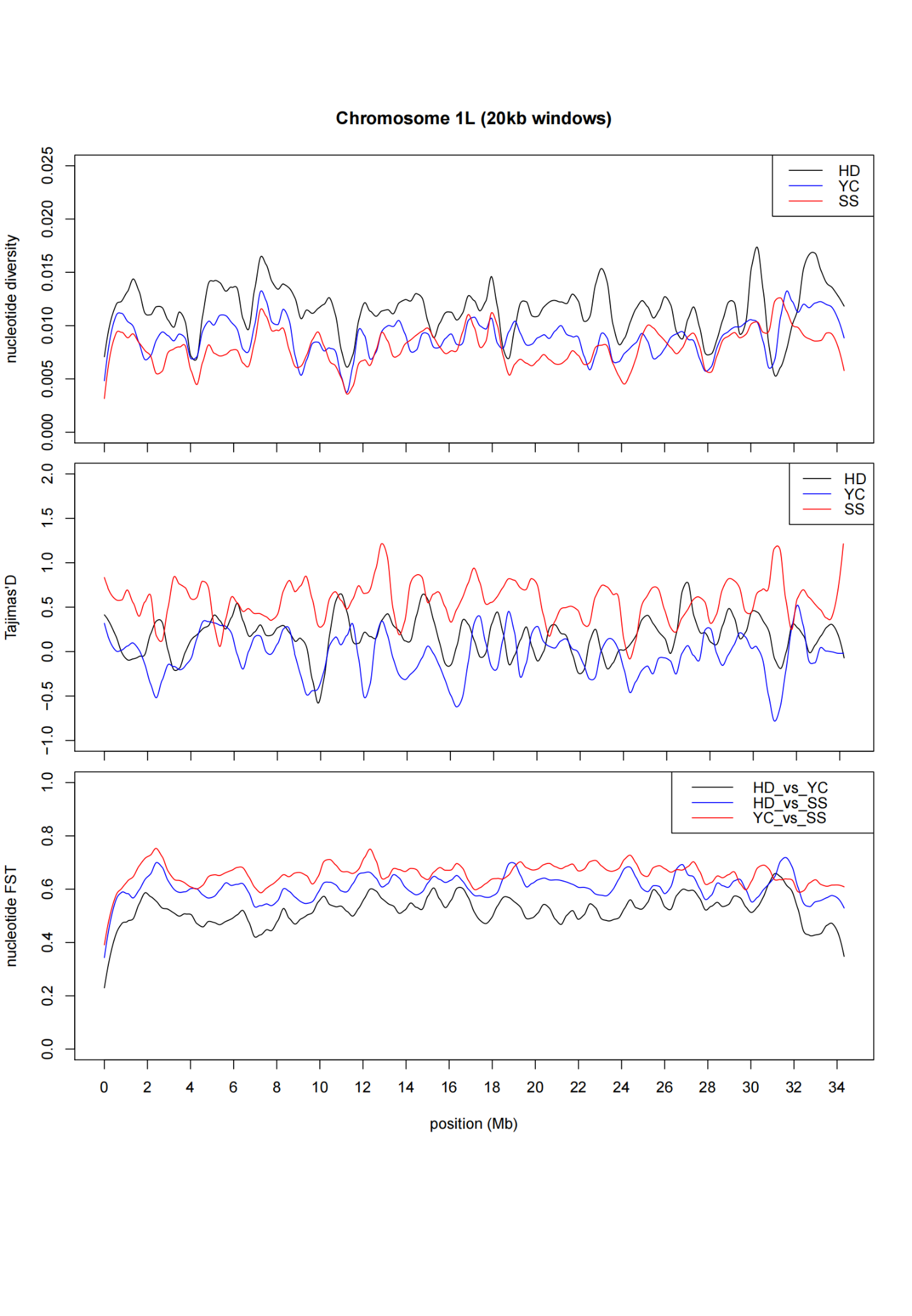


**Figure S13.** Overlapped sliding window analysis of nucleotide acid polymorphisms, Tajima’s D and genetic differential index with window size of 20kb and step of 2kb across pseudochromosomes 1 for different geographic populations of *Barthea barthei.*


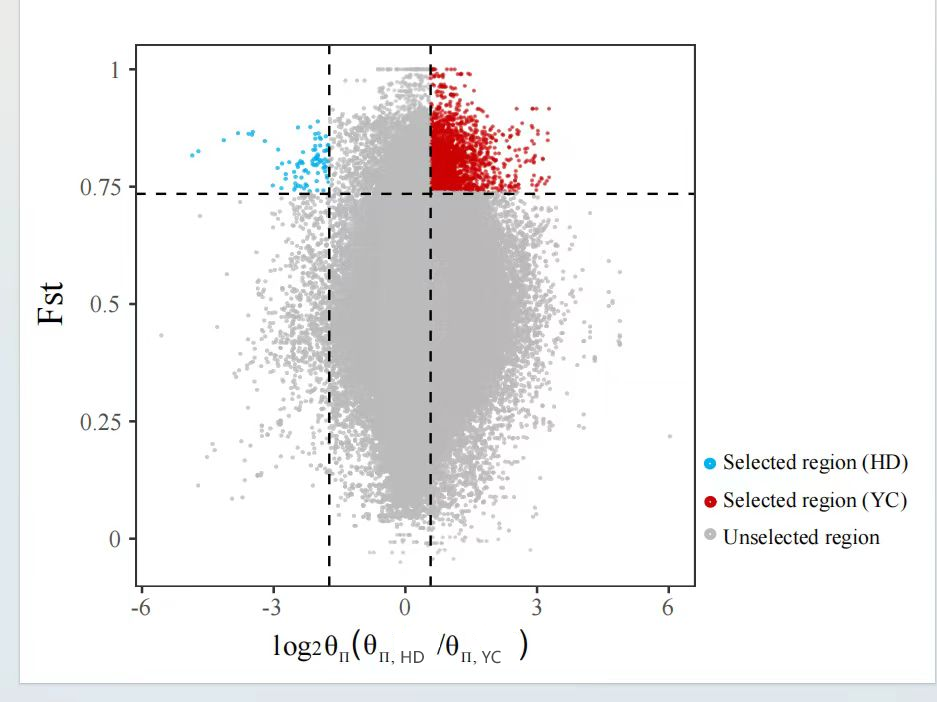


**Figure S14.** Genome wide windows under positive selection jointly determined by the distributions of nucleotide acid polymorphism ratio and genetic differential index (F_ST_) between HD population and YC population of *Barthea barthei* using overlapped window size of 20Kb and step size of 2Kb. Red and blue dots represent windows fulfilling the selected regions requirement.
